# A Practical Treatise on Venereal Diseases

**Published:** 1840-10

**Authors:** 


### Art. V.

1. *Traité pratique des Maladies Vénériennes; ou Recherches critiques et expérimentales sur l'Inoculation appliquée à l'étude de ces Maladies.* Par Ph. Ricord, Chirurgien de l'Hôpital des Vénériennes de Paris.—*Paris,* 1838. 8vo, pp. 808.
*A practical Treatise on Venereal Diseases, or critical and experimental Researches on Inoculation applied to the study of these Affections.* By Ph. Ricord.

2. *Recherches pratiques sur la Thérapeutique de la Syphilis, ouvrage fondé sur les observations recueillies dans le service et sous les yeux de M. Cullerier, Chirurgien en Chef de l'Hôpital des Vénériennes.* Par Lucas Championniere, Docteur en Médecine.—*Paris,* 1836. 8vo, pp. 415.
*Practical Researches on the Therapeutics of Syphilis, founded on observations gathered from the practice of M. Cullerier, Senior Surgeon of the Civil Venereal Hospitals.* By Lucas Championniere.—*Paris,* 1836.

3. *A Treatise on the Venereal Disease and its Varieties.* By William Wallace, Surgeon to the Jervis Street Infirmary, Dublin, &c.—*London,* 1838. 8vo, pp. 382.

4. *A Treatise on Syphilis.* By Herbert Mayo, F.R.S., Senior Surgeon of the Middlesex Hospital.—*London,* 1840. 8vo, pp. 128.

5. *The Modern Treatment of Syphilitic Diseases, both primary and secondary, containing an account of the New Remedies, with numerous formulæ for their preparation and mode of administration.* By Langston Parker, Lecturer on Anatomy and Physiology in the Birmingham Royal School of Medicine.—*London,* 1839. 12mo, pp. 158.

6. *Report of Primary Syphilitic Cases.* By C. Aston Key. (*Guy's Hospital Reports, October,* 1839.)

7. *On the Venereal Cases in which Mercury is admissible or is not admissible.* (*Dublin Journal, March,* 1838.) *Clinical Lectures on the Venereal Disease.* (*Dublin Med. Press,* 1840.) By Richard Carmichael, Esq.

8. *Manuel pratique de la Maladies de la Peau, appelées Syphilides, d'après les Leçons Cliniques de M. Biett.* Par Jos. Humbert, D.M P. Ancien Chirurgien interne du Dispendiare de l'Hôpital Saint Louis. —*Paris,* 1833. 12mo, pp. 220.
*A practical Manual of the Venereal Diseases of the Skin, from the Clinical Lectures of M. Biett.* By J. Humbert, D.M.P.—*Paris,* 1833.

9. *A Treatise on the Venereal Diseases of the Eye.* By Wm. Lawrence, F.R.S.—*London,* 1830. 8vo, pp. 337.

10. *A practical Treatise on the Diseases of the Eye.* By Wm. Mackenzie, (*Chap. XII., containing the Syphilitic and Gonorrhœal Ophthalmiæ.*) —*London,* 1840. Third Edition.

11. *Observations on the Venereal Disease and Venereal Inoculation.* By W. ACTON, Esq. (*Lancet, Nov.* 30, 1839, *Jan.* 4, *March* 7, 1840.)

12. *Beiträge zur Lehre von den Syphilitischen Krankheiten.* Von Dr. HAUCK, Königl. Stabsartze am Charité-Krankenhause, Berlin. (*Rust's Magazin, Berlin,* 1840.)

*Contributions to our Knowledge of Syphilitic Diseases.* By Dr. HAUCK, of Berlin.

IN a former Number, (Br. and For. Med. Rev., Vol. V., p. 1,) we devoted considerable space to the consideration of syphilitic diseases. We there dwelt chiefly on the natural history of syphilis, and entered but briefly into the question of treatment: in the present article we propose to pursue more fully our enquiries into the therapeutics of syphilis, first generally, and then in reference to the particular forms of disease. Before entering, however, into the consideration of treatment, properly so called, it will be necessary to pass in review the important addition to our knowledge made by the researches of Ricord on Inoculation as applied to the diagnosis of venereal diseases.

It would carry us far beyond our limits to enter into the vexed question of the identity or non-identity of the venereal poison; whether all the affections succeeding to sores or discharges from the genitals are due to the application of one virus, whether they result from the action of many, or from one in different states of modification. We apprehend, however, that the facts we shall have to bring forward will go far to prove that if the different forms of venereal diseases, both in their primary and secondary varieties, are not owing to the absorption of poisons of a totally different character, they at least depend upon a virus very differently modified; and it is with surprise that we observe a writer like the late Dr. Wallace maintaining, at the present day, the identity of gonorrhœa and chancres, and recommending what must be considered in some measure an indiscriminate mercurial treatment for both.

M. Ricord informs us that he entered on his experiments on inoculation without preconceived notions or prejudices, and that he instituted them to determine the following points: 1. The existence of a special cause for the production of syphilitic diseases—the venereal virus. 2. To distinguish, one from the other, diseases apparently similar. 3. To establish the differences which exist between primary or local and secondary or constitutional syphilis. 4. To endeavour to frame a rational preventive and a curative treatment,—a preventive treatment for secondary symptoms, a curative for primary ones. 5. To settle some points in relation to hygiène and forensic medicine.

M. Ricord's experiments were performed by taking some of the secretion from the surface of a venereal sore, or the matter of gonorrhœa, upon the point of a lancet, and introducing it by puncture in the skin of the thigh of the patient himself.

In reference to the first question proposed, M. Ricord's experiments establish that chancre, whatever may be its seat, is the consequence of the application of a specific pus which a chancre alone secretes, which matter produces a similar chancre whenever placed in circumstances favorable to contagion. To repeat this experiment with success, it must be recollected that chancres are divisible into two distinct stages recognized by Ricord, Wallace, Carmichael, Evans, and others, viz. the stage of ulceration and that of reparation or granulation. It is during the first stage only that the chancre secretes a specific pus, capable of propagating the disease, and producing a similar sore by inoculation : when the sore begins to heal, when granulations spring up, covered by simple puriform matter, the disease is not capable of propagation. In this case inoculation is negative and produces no result, from the circumstance that the sore no longer furnishes a specific matter, but simple pus not varying in its character from that of the secretion of an ordinary sore whose origin is due to other causes.

Mr. Evans states that from his experiments he is led to conclude that the fluid secreted by a chancre varies in its effects. He proves that the earlier the infection is taken, whilst the sore is in its excavated or ulcerating state, and, as we may infer, before the matter is truly purulent, the more severe and obstinate is the ulcer which it produces. The experiments of M. Ricord have been repeated by Dr. Mairion at the military hospital of Louvain, on 228 cases of various forms of syphilis: out of these 228 cases eighty-five were chancres or primary venereal sores, and of these eighty-five, fifty-three furnished the characteristic pustule by inoculation. M. Ricord describes the pustule succeeding to inoculation with the matter from a true chancre as follows (we avail ourselves of Mr. Parker's translation):

"If matter be taken from a chancre during the period of ulceration, and introduced under the epidermis by means of a lancet, it produces the following effects: During the first four and twenty hours the puncture becomes more or less inflamed; from the second to the third day it is accompanied with a slight tumefaction, and presents the appearance of a small papula surrounded with a red areola; from the third to the fourth day the disease assumes a vesicular form, the epidermis being raised by a fluid more or less opaque, presenting at its apex a small dark point; from the fourth to the fifth day the contents of the vesicle become purulent, the apex of the pustule is depressed, resembling very much the pustule of smallpox. At this period the areola, which had progressively increased, begins to diminish or altogether disappears, particularly if the disease does not increase; after the fifth day, however, the subjacent and surrounding tissues, which hitherto had undergone little or no modification, or were merely slightly œdematous, become indurated by the extravasation of a plastic lymph, which communicates to the touch the resistance and elasticity of cartilage. After the sixth day the contents of the pustule thicken, the pustule itself shrivels up and is covered with crusts. These enlarge towards their base, and forming by successive strata, at length assume the form of a truncated cone with a depressed apex. If these crusts are detached or if they fall off, we find under them an ulcer with a hard base, extending through the whole thickness of the skin. The surface of this ulcer, of a deep red colour, is foul, covered with a thick adhesive pultaceous matter almost like a false membrane, which cannot be removed by any attempt to clean the sore. The edges of the ulceration at this period appear as though it had been dug out from the surrounding parts by a sharp circular instrument. The immediate vicinity of the sore is surrounded by a dark red or livid margin, more elevated than the surrounding parts." (Parker, pp. 31-2.)

M. Ricord gives 389 cases of chancres which, when tested during the period of ulceration, produced this characteristic pustule by inoculation. In the cases detailed by M. Mairion thirty-two ulcerations upon the genitals out of eighty-five did not yield the characteristic pustule, the punctures being followed merely by a slight degree of inflammation which generally disappeared in the course of twenty-four hours. These then were cases of simple ulcers and not true chancres. It is evident that the experiments of Ricord prove beyond the possibility of doubt the existence of a true "venereal virus," a specific morbid poison. Dr. Wallace, without being aware of Ricord's experiments, states as a characteristic of the legitimate form or primitive type of true syphilis, "that it is more easily propagated by inoculation than any of the other forms of the venereal disease." This is owing to its independence of contingent or accidental circumstances; and hence it is probably the form of primary syphilis which would invariably occur if the action of the poison was uninfluenced by any such circumstances; whereas we often fail in producing the other forms of venereal diseases because they are partly the result of accidental causes which may not be present at the time of inoculation. In Dr. Wallace's clinical lectures, published in the Lancet, many experiments are detailed which entirely corroborate the statements of Ricord. He says (vol. ii., 1836-7) "the local specific effects which result from inoculation with the matter of the primary pustule commence almost immediately. In all the experiments which I have detailed, specific inflammation was produced within the second day, and in three or four days the characteristic pustule was fully developed." Both Carmichael and Mayo corroborate the accuracy and value of Ricord's experiments.

The mixture of the true venereal virus, secreted from a chancre in the state of ulceration, with the ordinary mucus of the vagina, the urethra, or the surface of the prepuce, or with the secretions of these parts in a state of inflammation, does not destroy its specific character or prevent it producing the characteristic pustule by inoculation, unless it be too much diluted. Various chemical agents, however, destroy its powers, and it no longer produces any result when tested by inoculation. Chemical substances decompose it. Amongst these Ricord mentions alkalies, and moderately concentrated acids, as the nitric, hydrochloric, sulphuric, and acetic; potass, soda, ammonia, wine, alcohol, and a strong decoction of tan: the active principle of the latter with wine forms, as we shall presently see, one of his favorite applications to primary syphilitic sores.

The next question to be resolved is the identity or non-identity of the morbid poisons producing a true chancre and gonorrhœa. We believe the diseases to be totally distinct, as the experiments of Ricord and many others seem to us incontestably to prove. Not only are they proved to be distinct by the results drawn from inoculation but by their pathology and the effects of remedies. It is well known that Hunter considered them identical. Dr. Wallace maintains nearly the same views, considering gonorrhœa as a form of primary syphilis which he describes under the term "catarrhal primary syphilis;" and we find some writers even at the present day still maintaining that gonorrhœa gives rise to chancres followed by secondary symptoms: these discrepancies, however, we hope to succeed in some measure in reconciling. Gonorrhœal matter, says Ricord, applied to a healthy mucous surface produces a gonorrhœa, the more easily as the matter itself is more purulent and less mucous or sanious.

"In no instance is it capable of producing a true chancre, but as a simple irritant, like the fluid of coryza, for example, it may excoriate the skin with which it remains some time in contact, but cannot produce a specific ulcer. Convinced of these truths, so frequently verified, my pupil, M. Leon Ratier, has frequently inoculated the skin of his arm with gonorrhœal matter without the slightest result. The diseases consecutive to gonorrhœa do not furnish a specific pus or one producing a specific ulcer from inoculation. Constitutional syphilis does not succeed to gonorrhœa. Where these symptoms are said to have succeeded a gonorrhœa, in the observations recorded by authors, the cases are in a direct ratio of frequency with those of chancres of the urethra. These cases have been incorrectly diagnosticated, the diseased surfaces not having been explored." (Ricord, p. 133.)

In Mr. Acton's valuable papers in the Lancet, referred to at the head of this article, several interesting facts bearing on these points are recorded; and many others have been made public by other followers of M. Ricord.

Dr. Mairion tested eighty-five cases of gonorrhœa; of these, four yielded a specific pustule from inoculation—there were chancres of the urethra. The remaining eighty cases of simple gonorrhœa produced no result, however frequently the diseases were tested, which they were in all periods of their course. In one case the results were not noted. Mr. Carmichael, in his lectures on venereal diseases, recently published in the Dublin Medical Press, has misunderstood and consequently mis-stated some of Ricord's experiments. Mr. Carmichael there adduces some cases of discharges from the urethra which being tested by inoculation yielded the characteristic pustule of chancre. Mr. Carmichael gives this as a case of gonorrhœa; but Ricord distinctly calls it a case of concealed chancre, chancre of the urethra (chancres larvés.) "In pressing the fossa navicularis," says M. Ricord, "we felt the induration indicating the seat of the chancre." This is the case of B—r, quoted by Mr. Carmichael from p. 235 of Ricord's book as a case of gonorrhœa. The arguments generally adduced for the identity of the two poisons in chancre and gonorrhœa are, the occurrence of secondary syphilis after diseases characterized by discharges from the urethra only, and the fact that a female with an ulcer only shall produce gonorrhœa in one instance and a true venereal pustule in a second. The true conditions of these cases are well stated by Mr. Babington:

"With regard to the first of these," he says, "the instances are so rare that they must rather be considered as anomalies which we cannot yet account for than be admitted in contradiction to the general current of experience. The secondary symptoms in such instances are rarely of an indubitable character. It may be doubted whether distinct copper tubercles ever followed simple gonorrhœa. We have generally a mottled state of the skin, or the lighter and more fugitive forms of lichen, or slight excoriation of the surface of the tonsil. . . . . It has been asserted that women who are affected with gonorrhœa alone will frequently produce chancres in those men who are connected with them. Without venturing to give a positive denial to this statement, it may be suggested that its truth has seldom, if ever, been satisfactorily ascertained. The examination of the surgeon has evidently been confined to the external parts. The interior of the vagina has not been examined; yet it is certain that chancres do occur here, though more rarely, so as to be discovered only by the use of the speculum." (Babington, in Hunter's Works, pp. 147-8.)

Much confusion exists in Mr. Judd's account of the pathology of gonorrhœa, and he has evidently included diseases of a totally different character under the term "urethritis venerea." "Generally," says Mr. Judd, "gonorrhœa produces a discharge of pus only; but at other times it also produces a pustule or abscess within the urethra, similar to what it does more usually on the surface, and just like the discharge from a pustule on the glans penis." These latter are clearly examples of chancres seated within the urethra, whose existence has lately been so well pointed out by Ricord and Cullerier. Chancres seated within the urethra are not accompanied by the usual symptoms which characterize gonorrhœa. We have now under our care a gentleman who has a chancre extending for an inch or an inch and a half along the urethra; this gentleman has recovered rapidly under the influence of a mercurial course, to which he submitted himself without consulting a surgeon: the chancre of the urethra was accompanied by one on the prepuce, for which he adopted this plan of treatment, having before had chancres cured in this way. The chancre in the urethra, which at first attracted little attention on account of insensibility, healed with that on the prepuce. The discharge from the urethra was comparatively trifling, much less than in ordinary gonorrhœa, and the pain in micturition much less. Mr. Mayo mentions a case similar to this (p. 39), in which the patient had a chancre and a discharge from the urethra: he tested the matter from both by inoculation; to his great surprise the characteristic pustule of chancre succeeded in both instances; on pressing the lips of the urethra apart a chancre was discovered, and the result at once explained.

"I entertain little doubt that the various cases reported by so many, of the same woman infecting indifferently either with chancre or gonorrhœa, admit of the same explanation. The person who has communicated the two diseases has had them both. M. Ricord has established the fact that chancres often exist deep in the vagina, and even upon the os uteri. . . . . I believe, from observation, that such chancres, not external in women, may remain for months in an indolent and unprogressive state. I attended a gentleman for three successive chancres, which he had caught at intervals of a very few months from the same woman, who would have it she was in perfect health. At last she consented to allow me to examine her, when I found two small ulcers within the external labia, which got well under mercury." (Mayo, p. 40.)

The researches of M. Ricord on the nature and differential diagnosis of buboes are of equal interest. According to this author, buboes are of two kinds, simply inflammatory, or virulent: in the first instance, succeeding to gonorrhœa, balanitis, or any other primitive affection; and in the second, arising from the consequences of the direct absorption of specific matter from a chancre.

"The virulent bubo, or that resulting from the absorption of the specific pus from a chancre, is a disease precisely similar to chancre in its nature, differing from it only in its seat. The virulent bubo is the only form capable of propagation by inoculation. The symptoms hitherto indicated by authors, with a view of establishing a differential diagnosis between the virulent bubo and one merely inflammatory, are of little value, inoculation being the only certain and pathognomonic sign." (pp. 150-1.)

There is a species of bubo which is termed by the French writers "bubon d'emblée," that is, an enlargement and suppuration of one or more of the inguinal glands which has not been preceded by any other of the more common forms of venereal diseases, nor in fact by any other symptom. The existence of these buboes is also admitted by Fallopius, Astruc, Swediaur, Bertrandi, and lately by Dr. Mondret, Mr. Judd, and Mr. Parker. M. Ricord insists that when these buboes occur, without the intervention of any antecedent form of disease, it is impossible to judge of their true character without the test of inoculation, and, consequently impossible to heal them rationally or well. M. Ricord maintains that those only which furnish the true characteristic pustule of chancre by inoculation are those only which are capable of being followed by secondary symptoms. Mr. Judd mentions a considerable number of cases of this kind, where buboes succeeded to connexions with prostitutes without any other symptom of syphilis having preceded them. None of the parties mentioned by him had any other affection to which the buboes could be traced; and in one case the affection was clearly venereal, for it was followed by nocturnal pains, and a true syphilitic eruption of mottled skin, or exanthema roseolum. In testing open buboes, M. Ricord impresses upon us the necessity of taking the matter for experiment from the secreting surface of the sore itself, and not from the pus which may run out from the bubo, since he constantly found that the latter, which was doubtless ordinary pus, produced no effect, whilst the former—that from the surface or bottom of the sore itself—was followed, where the bubo was venereal, by the usual characteristic pustule of chancre.

Hunter denied the power of secondary syphilis to be propagated by inoculation, and the modern researches of Ricord have corroborated all that Hunter taught and wrote on this point. The virus absorbed from a chancre may produce the characteristic pustule, and thus propagate the disease by inoculation, if the matter be taken from the surface of the chancre itself during its period of ulceration, or from the lymphatic vessel or vessels leading from the chancre to the first lymphatic gland, or from the gland itself should this inflame and suppurate; but beyond this point where the virus becomes mixed with the blood, most probably through the medium of venous absorption, the disease is not capable of being propagated by inoculation, nor do secondary affections, properly so called, such as ulcers of the throat or skin, or ulcerating tubercles, or any of the forms of secondary or constitutional syphilis, produce venereal sores when tested by inoculation. M. Ricord concludes from his experiments, which perfectly establish those of Hunter,

> "That when we find the forms of secondary syphilis not transmitted by inoculation, it is not because the disease is no longer syphilitic, but that the virus, modified by its mixture with the blood, loses the power of being propagated by inoculation, and at the same time acquires another, that of being transmitted by hereditary taint. That whenever a syphilitic sore, whatever may be its seat and form, produces a chancre by inoculation, it is of necessity a primary affection, and not a secondary or constitutional one." (pp. 166-7.)

We are informed by Sir George Ballingall, that "in the Lock Hospital at Edinburgh, the experiments of Ricord upon inoculation from primary sores have been lately repeated, and with pretty nearly the same results. One of the principal points ascertained by them is that chancres of a true syphilitic character, according to Ricord's test, may heal under the simplest treatment in the course of a few days, and consequently that the shortness of time an ulcer upon the genitals remains open does not furnish, as has been sometimes supposed, a proper criterion of the non-syphilitic nature of that ulcer." (Ballingall's Outlines of Military Surgery, 2d Edit., Edinb. 1838, pp. 419-20.)

There are certain circumstances, however, in which secondary symptoms are propagated by inoculation, as from the mouth of an infant having sores from constitutional syphilis transmitted by hereditary taint to the nipple of its nurse. In these instances the nipple of the nurse becomes ulcerated, and the ulcer "will not resemble the fissures which are so common on the nipple of women who give suck, and which occasion no loss of substance, but will be a corroding ulcer, and will destroy the whole or greater part of the nipple before it is healed." (Babington, in Hunter, p. 476.) From these ulcers on the nipples the glands in the axillæ frequently enlarge, and, sooner or later, if remedies be not used, sore throat, eruptions, or nodes arise, which are not distinguishable from the ordinary forms of constitutional syphilis.

These facts are the chief exceptions to the principles laid down by Hunter and Ricord of the non-transmission of secondary syphilis by inoculation, and "it is difficult in the face of them to deny that they are the effect of the venereal virus."

Some experiments detailed by Dr. Wallace in the lectures before quoted, seem in some measure to invalidate the statement that secondary symptoms are not capable of being propagated by inoculation. He shows that inoculation with the matter from a constitutional pustule very often fails to produce a specific effect. So far Dr. Wallace agrees with the opinions of Hunter and Ricord; but he adds that it does occasionally succeed, not in producing a sore having the character of a true chancre, but one resembling the ulcers which accompany the constitutional forms of syphilis. Dr. Wallace observes that inoculations with this matter succeed much more frequently if applied to a surface than if introduced by puncture. Dr. Wallace's experiments and remarks certainly prove that the laws which regulate the propagation of the two forms of disease are very different. Dr. Wallace compares them to the modes in which scabies and vaccination are propagated, one by contact, the other by puncture.

We now proceed with our enquiry into the modern treatment of syphilis. We need not here enquire into the comparative merits of the two opposite modes of treating the affection practised at the present day, or detail the statistical results of such treatment: for these we refer our readers to the comprehensive article in our Ninth Number. The treatment of syphilis without mercury is termed "the simple or physiological method," the treatment with mercury "the revulsive or mercurial." We purpose in the remainder of this article to confine ourselves to the question of the actual mode in which the modified mercurial treatment is carried on by modern surgeons, referring our readers to our former article for details of the simple treatment.

I. Primary Syphilis. We shall commence with the catarrhal affections. There is a true and false gonorrhœa properly so called; and also gonorrhœal diseases of the eye. This class of affections constitutes the non-virulent venereal diseases of Ricord, the catarrhal primary syphilis of Dr. Wallace, and the erythematous primary diseases of M. Desruelles. The first affection of this class is that described by

M. Desruelles under the term "balanitis," the external, bastard, or false gonorrhœa of Ricord. This affection consists in inflammation, with a muco-purulent discharge, from the mucous membrane covering the glans penis. When the disease extends to the under surface of the prepuce it is called balano-posthitis, and this is its most general form.

"The symptoms which ordinarily denote the existence of balanitis are heat and itching of the glans penis and prepuce, with a discharge of variable character from the orifice of the latter: these symptoms may be accompanied by phymosis or paraphymosis. When the prepuce can be drawn back and the glans uncovered, this is found red, swollen, and covered with a muco-purulent fluid of an unpleasant smell. The epithelium of the glans and prepuce is detached in places, excoriated, but not in a state of true ulceration. The testes and glands of the groin are more or less swollen and tender; we have seen the latter occasionally suppurate and bubo supervene upon balanitis." (Parker, p. 41.)

Balanitis may be the consequence of connexion with women suffering from gonorrhœa, but more commonly succeeds to intercourse with females menstruating, labouring under leucorrhœa, simple inflammatory affections of the vagina, or when this part is covered with secretions of a more or less irritating character. Mr. Judd gives several cases where a species of bastard gonorrhœa, soon subsiding, succeeded intercourse with females during menstruation. In some instances, balanitis occurs to married men who have had no intercourse, except with their wives, and where there has not been the least reason to suspect the character of the female. Mr. Parker relates a very remarkable case of this kind:

"A lady of most irreproachable and exemplary character, the mother of nine children, in the seventh month of her pregnancy of her tenth child, became affected with itchings and swelling of the labia, and a muco-purulent discharge from the vagina: her husband consulted me a few weeks afterwards, having certainly had no other connexion, with severe inflammation and excoriation of the surface of the glans and prepuce, from which oozed a muco-purulent fluid. Some slight astringent washes soon removed the disease, which was thought of no more. The lady, however, became again pregnant, and about the same period of her pregnancy her leucorrhœa again returned more severely than before. Her husband again consulted me: the internal surface of the prepuce and glans were swollen, intensely red, and painful, and covered with small aphthæ; in some places the mucous membrane was denuded, exposing a deeply-red surface secreting a thick pus." (Parker, p. 81.)

It is of great importance, where the character of a female is at stake, to determine whether the affection she is labouring under be merely a chronic or subacute vaginitis, or a true gonorrhœa. This point engaged the attention of Mr. Hunter. "Gonorrhœa," says he, "is not so easily known in them as in men, because the parts commonly affected in women are very subject to a disease resembling the gonorrhœa, called fluor albus; and the distinguishing marks, if there be any, have not yet been completely ascertained. The kind of matter gives us no assistance in distinguishing the two diseases, for it often happens that the discharge in the fluor albus puts on all the appearance of the venereal matter; and an increase of discharge is no better mark by which we can distinguish the one from the other. Pain, or any peculiarity in the sensation of the parts, is not a necessary attendant upon this complaint in women, therefore not to be looked for as a distinguishing symptom." (Op. cit.

pp. 186-7.) Modern authorities do not seem to have advanced much since the time of Hunter in determining the differential diagnosis of the two diseases. "The diagnosis of leucorrhœa," says Dr. Churchill, in his Outlines of the Principal Diseases of Females, "is, according to all authorities, extremely difficult." Sir C. M. Clarke seems to think it impossible. Ricord thinks all doubt may be removed by an examination with the speculum. Whenever the peculiar erosions or superficial ulcers of the mucous membrane covering the cervix uteri are discovered, and which occur in nineteen out of twenty acute cases, we can have no hesitation in pronouncing the disease to be true gonorrhœa. Uncomplicated balanitis generally gives way quickly to remedies of an appropriate kind. If the prepuce can be denuded, Ricord recommends the surface of the glans and prepuce to be superficially cauterized, by drawing the solid nitrate of silver quickly over the whole diseased surface. Slightly astringent injections between the glans and prepuce are of great service, as recommended by Desruelles and Cullerier. Mr. Parker recommends the following liniment to be introduced between the glans and prepuce by means of a camel-hair pencil: ℞. Cerati simp. v. Mellis, Olei Olivæ, āā ℥j. Hydrarg. Chlorid., ʒss. P. Opii, ʒj. M. All the modern writers under review agree in the propriety of an antiphlogistic treatment in the early stages of acute or virulent gonorrhœa.

"In the early stage of the more phlegmonoid varieties, and these occur in more healthy habits, great advantage will be obtained in the first instance by such remedies as subdue vascular action, such as bloodletting, particularly local bloodletting, by the application of leeches to the region of the frænum, or by the division of one or more of the veins in the body of the penis; also by the gentle but full evacuation of the bowels, by antimonials, by perfect quietness, particularly in the recumbent posture, by abstinence, by fomenting the perineum with tepid water, by a very soft poultice to the end of the penis or orifice of the urethra, when the prepuce is not sufficiently long to cover the latter, by suspension of the testicles, by diluting, alkaline, and mucilaginous drinks, and by the use of the hip or general tepid bath." (Wallace, p. 256.)

When the acute inflammatory symptoms have been subdued, Dr. Wallace submitted his patients to the use of what may be termed the specific remedies in gonorrhœa; such are cubebs and the copaiba balsam, with injections of the nitrate of silver and mercury. Mr. Judd, Ricord, Cullerier, and Mr. Parker have employed the nitrate of silver injections with great benefit in gonorrhœa; there appears, however, amongst these writers great difference of opinion as to the degree of strength in which the solution of this salt should be employed. Dr. Wallace and Mr. Judd have used it in the proportions of fifteen grains or even more to the ounce of distilled water; Ricord and Mr. Parker recommend two grains to the half-pint. Sometimes the solution of nitrate of silver cannot be borne or does not check the discharge. In these cases the authors before us have proposed a vast variety of remedies. A favorite injection with M. Ricord is a solution of the ioduret of iron in the proportions of three grains to six ounces of distilled water. In weak solutions the ioduret of iron has frequently arrested the discharge in five or six days; again it has produced an acute attack of urethritis; but when this has subsided the patient has found himself cured of his gonorrhœa. Sir George Ballingall has made some observations on the use of the nitrate of silver injections, of the strength recommended by Dr. Wallace and Mr. Judd, and the results would lead us to make use of it with a less proportion of the salt. Sir G. Ballingall employed it in two cases, and both recovered quickly, but at the expense of much suffering. In twenty cases, in which it was used in the 88th regiment in the strength of a scruple to the ounce, the cases were cured at various periods from ten to forty-two days, the average length of time having been seventeen days. In seventeen cases treated in the same regiment by rest and abstinence, the average duration of the treatment was only eight days. Rest and abstinence, however, are not always to be enforced in private practice; and Sir G. Ballingall thinks that unless injections be employed immediately on the subsidence of the inflammatory symptoms, the disease may be protracted for months. Where the solution of the ioduret of iron has been borne, the average duration of the treatment has been seven or eight days. Mr. Judd has given from his own practice a table of the comparative powers of different injections in curing a gonorrhœa quickly.

| *Composition of the Injection.* | *No. of days in curing.* |
|---|---|
| Sol. Liq. Plumbi, c. Ext. Belladonnæ. | Two. |
| Ditto. | Five. |
| Ditto. | Three. |
| Ditto. | Five. |
| Tinct. Ferri Mur. c. aquâ. | Five. |
| Ditto. | Four. |
| Ditto. | Seven. |
| Tinct. Ferri Mur. c. aquâ. Sp. Cubebæ et Copaibæ. | Six. |
| Sol. Argenti Nitratis. | Five. |
| Ditto, et Copaibæ. | Seven. |
| Ext. Cubebæ. | Three. |
| Ess. Cubebæ et Bals. Copaibæ. Inject. Zinci Sulphatis. | By twice using in one evening. |
| Ditto. | One. |

All these persons, says Mr. Judd, were cured in unusually short periods, of from one to seven days, and the last two only appeared to suffer from the sudden suppression.

The formulæ for a great variety of injections are given by Mr. Parker, (pp. 65-9.) Amongst these a favorite one of M. Ricord may be mentioned, consisting of a solution of pure tannin in port-wine in the proportions of eighteen grains of the former to six ounces of the latter. This is occasionally very useful. We have known a few grains of tannin placed upon the tongue quickly cure a salivation which no other remedy would check. Dr. Wallace employed mercury, given internally, in gonorrhœa, or what he terms catarrhal primary syphilis. The grounds upon which this is recommended are certainly not sufficient to warrant the general exhibition of this remedy in the ordinary forms of gonorrhœa, and we had thought the experience of modern surgeons had set this matter at rest. No British surgeon can forget the manly manner in which Sir A. Cooper exposed the abominable system of submitting the patients in Guy's Hospital to courses of mercury for the cure of gonorrhœa. "To compel an unfortunate patient to undergo a course of mercury for a disease which does not require it is a proceeding which reflects disgrace and dishonour on the character of a medical institution." Yet we find Dr. Wallace, on the theoretical assumption of the identity of the poisons in gonorrhœa and syphilis, recommending five grains of blue pill with a quarter of opium twice a day for two or three weeks. "This," says Dr. Wallace, "has appeared to me sufficient to afford all the assistance required in general for the removal of the disease, or to protect the constitution from contamination." What has been already stated respecting masked or urethral chancres will explain the favorable results obtained by Mr. Wallace in some cases, and account for such a practice having been adopted by so experienced a surgeon.

Although gonorrhœa is not followed (except in very rare instances, and even these are matters of doubt) by secondary symptoms, properly so called, it appears to dispose the economy, either from sympathy, metastasis, or the actual contact of gonorrhœal matter to other mucous surfaces, to several diseases of a destructive character, which are of more importance than the affection to which their origin is due. Amongst these we shall here briefly notice diseases of the testicles and of the eye, as the works before us furnish some new and important facts on both these heads.

*Swelled Testicle.* The most frequent of all the diseases of the testicle which succeed to or complicate a gonorrhœa is inflammation of the epididymis, and so frequent and regularly does this occur in many cases of gonorrhœa, that Ricord terms it "blennorrhagic epididymitis." This disease is seated in the convolutions of the epididymis and not in the substance of the testicle itself. It has generally been supposed that this affection succeeds to the sudden suppression, or quick cure of a gonorrhœa; but M. Ricord thinks, and we believe with justice, that the chances of a swelled testicle are increased in direct ratio to the continuance of the disease. "The disease in question," he says, "does not happen once in 300 times in the first week of a gonorrhœa, commonly it occurs after the second week, but it is most frequent during the third or even at a later period; and this takes place both in chronic and acute cases." (pp. 749-50.) The occurrence of this disease is favoured during the continuance of a gonorrhœa by exercise, constipation, the abuse of stimulating drinks, and the neglect of the suspender. M. Ricord describes two forms of this disease: one in which the epididymis becomes inflamed from sympathy, without the inflammation having passed along the vas deferens, and a second in which the inflammation has advanced along the ejaculating duct to the vesicula seminalis, and thence along the vas deferens to the epididymis. If this species of inflammation occur with any degree of intensity, it soon involves the testicle and its envelopes, and hence, succeeding or complicating it, we commonly find inflammatory hydrocele, œdema, or phlegmon of the scrotum, with inflammation of the substance of the gland itself. Dr. Wallace remarks that this variety of diseased testicle "is distinguished by its occurring at the *seeming* decline of the original affection, and by the vas deferens being diseased before the epididymis, and the latter before the body of the testicle, and it will be found that in proportion as the original disease retreats, as it were from the anterior extremity of the urethra, the more deeply-seated portions of this canal become affected with tumidity and tenderness." These remarks favour also the opinion of Ricord, that the probabilities of the disease extending to the testicle are in direct ratio with the time of its continuance, and hence the early use of specific remedies, are our best guarantees against consecutive diseases of the testicle.

The treatment generally adopted in affections of this kind consists of general and local bleeding, fomentations, poultices, with evaporating and slightly astringent lotions. Under this plan patients are generally a long time in recovering, and its use confines them to bed and the recumbent posture. In reference to bleeding, both general and local, we perfectly agree with Dr. Wallace :

"In some cases general bleeding will be useful, but in the majority it will not be necessary. The inflammation is for the most part of an irritable character, and irritable inflammations are seldom benefited by venesection. The topical abstraction of blood by unloading the weak and distended capillaries, without debilitating the system, may be more useful, but the circulation in these vessels is in general relieved by position; and the patient should preserve a recumbent posture, with the testicles carefully suspended." (Loc. cit.)

Suspension of the testicles with gentle pressure is of great service in affections of this character, and has given rise to the treatment now uniformly practised by Fricke in Hamburg, and by Velpeau and Ricord in Paris. Compression in inflammation of the epididymis and the envelopes of the testicle generally effects a cure in four, five, or six days. It prevents the occurrence of hydrocele, and one inestimable advantage is that during its employ the patients can, except in very acute cases, follow their occupations. For a full account of this practice, as first introduced by Fricke, we refer the reader to the Second Volume of this Review, (p. 253.) Since that paper was published we have adopted the practice in many cases, and can speak most unequivocally in its praise; and it may be employed even in very acute cases. We have seen several instances where the patients have not lain in bed an hour, whilst under the ordinary antiphlogistic treatment they would have been confined from ten days to a fortnight.

*Venereal Diseases of the Eye.* Whilst in ordinary cases secondary symptoms, properly so called, do not follow a common gonorrhœa, the continuance of this disease for any length of time, particularly in irritable subjects, appears to dispose the economy to the attack of several diseases which are of a more formidable character in their results than the constitutional symptoms which succeed to primary syphilitic sores. Of this character are several diseases of the eye, and affections of the fibrous and synovial systems. The first and perhaps the most formidable affection of the eye we have to notice is that which is commonly termed gonorrhœal conjunctivitis. This disease frequently affects children, and is then termed ophthalmia neonatorum, or the purulent ophthalmia of infants. "There is reason to suppose," says Dr. Mackenzie, "that this disease is not unfrequently an inoculation of the conjunctiva by leucorrhœal fluid during parturition; and that therefore it may often be prevented by washing the eyes of the infant with tepid water, as soon as it is removed from the mother." (Practical Treatise on Diseases of the Eye, third edition, 1840.) The worst form of this disease is, however, the result of the direct application of gonorrhœal matter to the surface of the conjunctiva. One of the most frequent results of this most formidable disease is purulent infiltration of the cornea, " by which its texture is speedily destroyed, first of all exteriorly to the pus effused between its lamellæ, and then through its whole thickness, and this in a small spot only, or over its whole extent; so that sometimes we find only a small penetrating ulcer, with the iris passing through it; in other cases the whole cornea is gone, the iris exposed, and the humours protruding through the pupil." In many instances also the lens comes away. In recent cases the eyes are to be washed out every eight hours with a collyrium composed of one grain of the bichloride of mercury, and eight of the muriate of ammonia, in eight ounces of water, and immediately after this has been done, either with a soft sponge or by means of a syringe, a solution of the nitrate of silver in the proportion of ten grains to the ounce is to be applied over the whole surface of the inflamed conjunctiva. Under this plan of treatment the infant generally opens the eyes in two or three days, and in ten or twelve the acute symptoms are overcome. We have frequently seen acute recent cases of purulent ophthalmia cured in three or four days by introducing daily between the lids a portion of ointment composed of ten grains of nitrate of silver, twenty minims of the liquor plumbi diacetatis, and one drachm of adeps, or unguentum cetacei. With these remedies local depletion and blisters may in certain cases be used. "In cases which have been neglected for perhaps eight or ten days, it is necessary to take away blood from the external surface of the upper eyelid by the application of a leech, or from the conjunctiva by scarification. The former may be tried in the first instance, and unless followed by marked abatement of the redness and swelling on the inside of the lids the conjunctiva may next be divided with a lancet. The taking away of blood in either of these ways is productive of much benefit, and ought by no means to be omitted, if there be any tendency to chemosis or any threatening of haziness of the cornea." (Mackenzie, p. 40-3.) Blisters are very serviceable, particularly in sub-acute or chronic cases; the bowels should be opened in the acute forms by calomel and castor oil. In threatened disorganization of the cornea recourse must be had to the extract of cinchona or the sulphate of quina. In the relaxed states of the conjunctiva which succeed to the disease, the topical application of the vinum opii is useful. The pure gonorrhœal ophthalmia makes its appearance under three forms: 1st, It may arise from the direct application of gonorrhœal matter to the eye; 2dly, It has been supposed to be metastatic; and 3dly, It has been considered, at least in certain cases, as an effect owing to irritation merely without either the direct application of the matter or metastasis. Some writers have denied that the application of gonorrhœal matter to the eye of the same individual has power to produce the first form of the disease of which we are speaking, and this was the opinion of Dr. Vetch. Numerous cases, however, establishing the fact that the application of gonorrhœal matter produces the most destructive forms of inflammation of the eye have been detailed by Lawrence, Mackenzie, Wallace, Astruc, Foot, Wardrop, Delpech, and Bacot. In the second form of the disease the eye is said to suffer from metastasis; "it is stated that the gonorrhœal discharge is suppressed, and that the inflammation of the eyes occurs in consequence of that suppression." This view is supported by Richter, Scarpa, and Beer, and they consider the restoration of the discharge from the urethra as a principal indication in the treatment of the disease. Saint Yves speaks also of this disease, but neither Dr. Mackenzie nor Mr. Lawrence have met with cases of gonorrhœal ophthalmia from metastasis, neither does Dr. Wallace allude to it. The third form of disease, where gonorrhœal inflammation of the eye occurs during the continuance of a clap, without the direct application of the matter, or without its suppression, is admitted both by Dr. Wallace and Mr. Lawrence. "Since then gonorrhœal ophthalmia may occur whilst the discharge from the urethra continues, and since it does not take place when that discharge is stopped, we cannot admit that the affection of the eye owes its origin to the cessation of disease in the urethra. I am inclined to refer its occurrence to the state of the constitution, without being able to point out in what that state consists; and to regard it as a pathological phenomenon analogous to those successive attacks of different parts which are observed in gout and rheumatism." (Lawrence, p. 34.) Dr. Wallace is pretty nearly of the same opinion: "that this form of ophthalmia is not caused, like the preceding, by the direct contact of matter from without is demonstrated by the fact that it has been observed to occur more than once in the same individual, although every means had been most carefully employed to protect the eyes from contamination. Indeed I know several persons of the most cautious and cleanly habits who uniformly get this ophthalmia when they get a clap." (Wallace, p. 303.) Lawrence, Wardrop, and Bacot place their chief confidence in the treatment of the acute forms of this disease in "the boldest antiphlogistic treatment." Mr. Lawrence thinks "as much blood should be taken from the arm as will flow from the vein, and that the evacuation should be repeated as soon as the state of the circulation will allow us to get more." (p. 36.) Blood must also be taken from the temples by cupping and by the free application of leeches round the part until the pain and vascular congestion is relieved. Mr. Wardrop goes so far as to say that the only case of gonorrhœal ophthalmia he had seen in which the eye was saved was that of a young woman in whom venesection was repeated as often as blood could be got from the arm. Bleeding alone, however, must not be depended on, but at the very commencement of the disease local applications of an astringent character, hereafter to be mentioned, must be combined with it. Notwithstanding these authorities, we are disposed to think that bleeding has been too exclusively relied upon in this disease, which is in its commencement purely local; and Mr. Lawrence himself is dissatisfied with the results of the cases treated exclusively on this plan, although he attributes its want of success to its not having been employed to a sufficient extent. However, he mentions a case (Case 5) in which blood was taken very largely, both locally and generally, and other powerful antiphlogistic means were resorted to, yet the eye was lost. Mackenzie says "bleeding alone must not be depended on," and O'Halloran is of opinion that if any enquiry were instituted amongst army surgeons it would be found that those who had used the greatest depletion were the least successful practitioners." Ricord says we must on the onset bleed our patient largely from the arm, and apply leeches freely to the number of forty or fifty to the internal canthus, in the course of the angular vein; to the temple and along the course of the jugular of the same side. Desruelles and Cullerier follow nearly the same practice. Whatever plan is adopted it should be pursued with the greatest energy, as it is well known that vision may be irretrievably lost in forty-eight hours. "Not a moment should be lost in endeavouring to control the disease, and our treatment of catarrhal syphilis of the eye must be more energetic than in any other form of the disease." (Wallace, p. 302.)

Directly after the system has been depressed by the loss of blood we must have recourse to astringent or specific remedies. Amongst these the one entitled to most confidence is the solution or pommade of the nitrate of silver. Dr. Ridgway employed it in the proportion of ten grains to the ounce, dropped into the eye at the very commencement of the disease. O'Halloran "had become dissatisfied with the antiphlogistic treatment, from having found it frequently either insufficient or injurious, and was hence led to use astringents, not only in the early stage of the disease, but when the purulent discharge and chemosis were fully established. He employed the sulphate of copper in substance, rubbing with it the inner surface of the eyelids after everting them, or he dropped into the eye the ten-grain solution of the nitrate of silver." (Lawrence, p. 44.) Mr. Lawence says, "Destructive or injurious consequences have so frequently resulted under the usual management of this disease, that I should certainly employ the local astringent if I met with a case favorable to the trial; that is, where the affection had not extended beyond the conjunctiva." Mr. Lawrence subsequently mentions two cases where the astringent was successfully employed; in the latter after a large bleeding. (pp. 45-6.) Dr. Wallace recommends the application of the nitrate of silver after the abstraction of blood in sufficient quantity, "and when we have an opportunity of seeing the disease before the state of inflammation, this remedy should be employed without a moment's delay." After free depletion, Ricord recommends the eyelids to be everted, and the solid nitrate of silver to be passed over the surface of the palpebræ, and afterwards more superficially over the ocular portion of the conjunctiva; after this the surface of the eye is to be cleaned by a syringeful of cold water thrown over it, with a view of removing any portion of the salt that may remain upon the cornea. In the intervals of the dressings the eye is covered with a compress, soaked in a decoction of poppy-heads, and applied cold. M. Ricord recommends also the application of the extract of belladonna to the temples and round the orbits during the treatment, with a view of preventing any adhesions of the iris which commonly becomes affected, as well as other deep-seated structures of the eye. If the chemosis be great, Ricord recommends a portion of it to be removed with a pair of curved scissors, a measure of which Dr. Mackenzie speaks also in very high terms. If the inflammation do not yield speedily to large general depletions, M. Sanson, as we stated in our last Number, has resorted to the expedient of excising the secreting organs itself—the conjunctiva! He snips away with a pair of curved scissors all the salient portions of the membrane, and then cauterizes with the nitrate of silver the whole internal surface of both eyelids.

We think it perfectly useless, not to say criminal, in such cases, to waste the time, so precious to our patient, in administering the remedies looked upon as specific in gonorrhœa, as copaiva and cubebs, recommended by Dr. Wallace, and we are glad to find M. Ricord supporting this the true view of the matter. He says, "The anti-blenorrhœal remedies, properly so called, as copaiva and cubebs, have absolutely no action upon this disease, whatever may be their mode of administration."

We are not disposed to think very highly of the exhibition of mercury, so as to produce even its full action in this disease, and we think Dr. Wallace quite in error in giving it as an antisyphilitic. We have before shown the fallacy of Dr. Wallace's opinions on these points. Mr. Lawrence has seen gonorrhœal ophthalmia proceeding unchecked under the full mercurial action. Ricord expressly states that it is of no service, so also do Beer and Delpech. There is no objection to its use in the chronic forms of disease; but we lose time, and compromise the vision of the patient by placing any reliance upon it in the acute.

Those authors who support the view of gonorrhœal ophthalmia being produced by metastasis place great stress upon the restoration of the urethral discharge; it is also recommended in cases where this ceases during an attack of disease of this character. Swediaur, Richter, Beer, and Scarpa recommend the introduction of a bougie smeared with the discharge from the eye, or with gonorrhœal matter taken from another patient. In spite of the authorities of these names, we think their recommendation rather the result of preconceived theoretical notions than deductions from the results of treatment. Mr. Lawrence supports the latter opinion, and the modern writers of the greatest experience, as Ricord and Desruelles, agree with him. "If," says Ricord, "the discharge from the urethra is for a short time diminished, during an attack of gonorrhœal ophthalmia, it is never completely suppressed, and we can affirm, in spite of contrary opinions, that not the least benefit is to be expected from attempting to restore or increase it." (p. 763.) Swediaur, however, appears to have been successful in curing some chronic cases of this character by the restoration of the urethral discharge; but we cannot find in any late writer, neither have we ever seen, a case supporting the efficacy of this treatment.

Dr. Wallace thinks that the ill consequences which so commonly follow gonorrhœal ophthalmia, such as bursting of the cornea and discharge of the humours of the eye, protrusion of the iris, &c. may be prevented by evacuating the aqueous humour on the plan recommended by Mr. Wardrop.

> "It has always appeared to me very probable that the sloughing and ulcerating processes, which so often attack the cornea in this form of ophthalmia, are owing principally to distension of the eyeball by effusion into its chambers, for sloughing or ulceration is not a usual consequence of catarrhal syphilis in other parts, unless when strangulation has occurred; and acting on this opinion, I have on several occasions, and apparently with very considerable benefit, adopted Mr. Wardrop's recommendation of puncturing the cornea to cause the escape of the aqueous humour, and the consequent diminution of the state of distension." (p. 302.)

We think these remarks very valuable, and the practice deserving repeated trials before it is finally rejected. Dr. Wallace's remarks are forcibly illustrated by Mr. Lawrence's sixth case, in which the eye literally burst from distension of this kind.

Mackenzie, Lawrence, Brodie, Ricord, and Wallace admit a true gonorrhœal inflammation of the iris. This generally occurs in scrofulous patients labouring under gonorrhœa or gleet. Sometimes it succeeds to gonorrhœal inflammation of the conjunctiva or the sclerotic, or occurs with that peculiar species of rheumatism which sometimes accompanies a gonorrhœa. It very commonly alternates with affections of the joints, and an acute attack of synovitis frequently cures or very much relieves the inflammation of the eyes. The frequency with which this species of disease succeeds to mild gonorrhœal ophthalmia, and the facility with which adhesions of the iris take place, render it necessary that in the various forms of gonorrhœal ophthalmia we should adopt Ricord's plan of keeping the pupil dilated by the external application of belladonna. "This affection of the eye is exactly the same as rheumatic inflammation of the sclerotic and iris occurring independently of gonorrhœa. Both this and the mild purulent inflammation of the conjunctiva are to be regarded as rheumatic affections of the organ excited by gonorrhœa; that is, they take place in individuals in whom this constitutional disposition is shown by inflammation affecting either the synovial membranes or the fibrous structures of several joints." (Lawrence, p. 57.) Dr. Vetch, as well as the authors already mentioned, has given cases of this disease. In one instance the gonorrhœa was well marked and violent, and was succeeded by a swelled testicle; rheumatic inflammation of the joints and of the external proper tunics of the eye followed. They terminated in an irregular and contracted pupil, some opacity of the capsule of the lens, adhesions between it and the iris, and a considerable loss of vision. Generally however, the prognosis is favorable and the disease very much more under the control of art than the more acute forms of purulent ophthalmia. "The gonorrhœal is generally more rapid in its progress than any of the other varieties of iritis, and is one of the most severe and formidable whilst it lasts; but it yields more promptly to decided treatment than any of the rest, and affords examples of perfect recovery, even when the aqueous chambers are filled with lymph." (Mackenzie, p. 476.)

The treatment must consist, in the onset, of general and local bleeding, suited to the urgency of the symptoms; calomel and opium, so as rapidly to affect the system, and the application of the extract of belladonna (Mackenzie). Our chief reliance must be on mercury united with opium and antimony, and if there exist a rheumatic state of the system, colchicum and turpentine will be useful (Wallace). Sir B. Brodie places great reliance on colchicum. As local applications, warm decoctions of poppy are generally most agreeable to the patient's feelings, on account of the intolerable pain that sometimes attends the disease. "When the inflammation is checked blisters may be advantageously employed, and the cure may be completed by the Plummer's pill, with mild aperients and regulated diet." (Lawrence.)

*Primary Syphilitic Sores.* The second class of these diseases are primary syphilitic sores, the "maladies vénériennes primitives à forme ulcérative" of Desruelles; the "affections virulentes" of Ricord. In investigating the therapeutics of this class of venereal diseases, we do not propose to enter into the particulars of the comparative merits of the mercurial and simple treatment, but simply to indicate that best suited to the different varieties of primary syphilitic ulcers. Though renouncing an indiscriminate mercurial treatment, most modern surgeons, more particularly those of this country, look upon mercury as the most powerful and certain therapeutic agent employed in a vast majority of both the primary and secondary forms of syphilis. "Is there any experienced senior of the profession who, having a son of eighteen or twenty, and that son having a chancre, that would treat him without mercury? No: there is not such an unnatural person." (Bell's Institutes of Surgery, p. 228.) Mr. Carmichael, instead of rejecting mercury altogether on the one hand, or coinciding with Sir C. Bell's sweeping dogma on the other, has wisely endeavoured to point out where mercury is admissible, and where it is not admissible in primary venereal sores. This seems also to be the aim of most modern writers on syphilis who have devoted particular attention to the enquiry. "Why," enquires Mr. Parker (p. 11), "is mercury to be employed in the treatment of primary syphilis? To hasten the cicatrization of the ulcer, and to diminish the risk of secondary symptoms." The first point is established by the practice of most modern surgeons, both British and foreign. Ricord, Desruelles, and Cullerier, all partisans of the simple treatment, recommend mercury when the sore is indolent, does not cicatrize under the simple plan, when its edges are hard and elevated, or the sore leaves behind it, in healing, an indurated cicatrix.

"If," says M. Ricord, "we calculate the cure of a chancre from the day the ulcer has cicatrized, without troubling ourselves about what may take place after, it will be sometimes apparently quicker by the simple treatment without mercury, and in the hospitals, the patients will be a less time under treatment; but if we date the cure at the period when all induration has disappeared, we shall find the difference enormously in favour of the mercurial treatment; the induration continuing in the first instance an indefinite period, and giving rise much more frequently to secondary or constitutional symptoms. I have recourse to mercury whenever a certain degree of induration accompanies a chancre, when it does not speedily cicatrize, or when the induration remains after its apparent cure." (pp. 578-9.)

From M. Bacot's summary it appears that secondary symptoms occur in the proportion of at least one in ten in those cases where no mercury has been given; whilst on the contrary, the proportion of such cases is only as one to seventy-five where that remedy has been employed. Mr. Carmichael admits that "mercury is an agent of great utility in some forms and stages of venereal diseases, when duly administered under sound pathological principles, and not blindly given as a specific." (Dublin Journal of Medical Science, No. 37, p. 118.) Mr. Carmichael exhibits it under the following circumstances: 1st. In cases of the simple primary ulcer, when this does not yield to rest, the antiphlogistic treatment, and astringent washes. After the third or fourth week, Mr. Carmichael gives mercury in alterative doses with the same views that he would prescribe it for any indolent ulcer which was not venereal. This ulcer, amounting to nine tenths of the cases we meet with in practice, is characterized by its want of induration and phagedæna. 2dly. When the papular and pustular eruptions become scaly, and obviously on the decline, about the fourth or fifth week, and not yielding to sarsaparilla or antimonials, mercury is given in alterative doses with sarsaparilla. 3dly. In iritis, so as to produce its full effects upon the system. 4thly. In cases of nodes with inflammation of the periosteum. In the phagedænic forms of primary syphilis, Mr. Carmichael believes mercury in all stages and forms of the affection to be a most deceitful and dangerous remedy. 5thly. For the primary sore, the Hunterian chancre, with hardened edges and base, the "chancre induré" of M. Ricord, and for the scaly eruption which attends it, as well as the deep excavated ulcer of the tonsils, nodes, and other symptoms belonging to this form of disease, mercury, in full doses, may be esteemed a certain and expeditious remedy. M. Ricord, admitting as he does the uncertainty in which we still remain with regard to the particular kind of sore in which mercury should be exhibited, remarks that in this form the surgeon who omits the full mercurial treatment, ought to be considered responsible for the constitutional symptoms which are almost sure to succeed it. The testimony of Mr. Guthrie is pretty much to the same effect in cases of ulcers which had "the characteristic of chancre." In these instances, without the use of mercury, local applications of various kinds seemed to have little effect upon the sores, and they remained open for various periods, but all very long. "If they were ulcers without very marked appearance, and did not amend in the first fortnight or three weeks, they generally remained for five or seven weeks longer; and the only difference in this respect between them and the raised ulcer of the prepuce was that this often remained for a longer period, and that ulcers presenting the true character of chancre required a still longer period for their cure—that is, from six to eight, ten, or twenty, and even twenty-six weeks, healing up and ulcerating again on a hardened base." In this form of ulcer, Desruelles also admits the superior efficacy of mercury. When we are certain that no manifest cause of irritation interferes with the cicatrization of the sore, and that still remains indolent, a general mercurial treatment must be employed. "In these cases no medicine is so advantageous as mercury, and so marked are its effects, that we are tempted almost to regard it as a specific." (Desruelles, p. 506.)

The universal tendency of modern experience, then, is in favour of the mercurial treatment of this form of disease, and although this as well as the other forms of primary syphilis may get well without mercury, still the cure is uncertain and prolonged, and generally leaves behind it a hardened cicatrix, prone to ulcerate again. It is doubly important to heal this, as well as the other primary forms of syphilis, speedily, since it is incontestably proved that the risk of secondary symptoms occurring is in direct proportion to the period a primary sore remains open; a primary sore closing with a hardened cicatrix is certainly not cured.

Mercury should not be given during the state of fever or local inflammation which may be present during the first days of venereal ulcers, nor till the patient is prepared for it by an appropriate regimen. It is also generally improper to use mercury during the ulcerating stage of chancre; it must be abstained from till the sore has become stationary and indolent, or till it assumes a disposition to heal. If mercury be employed during the ulcerating, irritable, or inflamed condition of a chancre, it will most probably increase the inflammation, or "excite a state of irritable or indolent action, after which the system will become quite insensible to ordinary doses of this medicine; and if under such circumstances larger doses of mercury be employed, a peculiar and complex state will most probably result, determined in its character by the combined influence of the disease, the remedy, and the constitution of the patient, a state in which mercury acts as a poison, or in other words, not only aggravates all the symptoms, but perhaps excites a new train of peculiar morbid actions." (Wallace, p. 109.) Mercury may be employed by friction, or fumigation, or the proto-chloride, the bichloride, the proto-ioduret, the cyanuret, or the deuto-phosphate may be employed internally, in different doses and under various forms of combination. As we have not time to notice these, we refer the reader to Mr. Parker's excellent little work, which contains a full account of these remedies, the indications for their employment, and their mode of administration.

*The Local Treatment of Primary Venereal Sores.* Scarcely any two modern authors agree in their classification of primary sores, and hence we shall speak of those alluded to in the works before us after their pathological character, and not in conformity with the particular division of each writer under review. In this point of view primary sores resolve themselves naturally into a few species to which all those described by authors may be referred. These are: 1st. The simple primary sore, characterized rather by its negative than positive characters, i. e. by the absence of induration, irritability, or inflammation. 2d. The irritable. 3d. Those characterized by an excess of inflammatory action. 4th. The indurated, or true Hunterian chancre, the induration being a primary feature of the sore, and not being produced by the injudicious use of local stimulating dressings, "by the use of which," says Desruelles, "any primary sore may be made to assume the indurated character." 5th. Those spreading by rapid ulceration, or covered by foul sloughs of various colour and appearance. This variety being the ulcerative or sloughing phagedæna of the authors before us.

It will be necessary before speaking of the local treatment of chancre to repeat what has been said on the division of primary venereal sores into two periods—the first that of ulceration, and the second that of separation or cicatrization; the local remedies adapted to the first of these states being inadmissible and generally hurtful to the second.

In the majority of cases of simple primary venereal sores, during the first period, the nitrate of silver is the best application, and amongst the writers before us, Carmichael, Ricord, Wallace, Key, Parker, and Mayo support this practice. Ricord and Carmichael both state that the sooner an ulcer which secretes a morbid poison capable of infecting the constitution is healed, the more likely is the constitution to escape contamination. "When, therefore," says Mr. Carmichael, "a patient applied to me with an ulcer in its first stage, while yet excavated and secreting lymph, I instantly endeavoured to destroy its entire surface by a free application of lunar caustic; and I observed that when the eschar separated, I had in general the satisfaction of finding a simple sore instead of a poisonous one." (Dub. Med. Press, No. 67.) If the sore be much inflamed, the caustic is not to be used till the inflammation has been subdued by rest, aperients, general bloodletting if necessary, abstinence, and the mildest topical applications, as bread and water, or the turnip or carrot poultice, the liq. plumbi diacet. dilut., &c. &c.; should the ulcerative stage be passed, and granulations have sprung up under the treatment, which is sometimes the case, the nitrate of silver is of course to be omitted.

"Whilst a chancre continues in the state of ulceration, the application of the nitrate of silver must be repeated, waiting for the separation of the eschar produced by its application, to ascertain clearly the condition of the sore before we reapply the caustic. After the application of the nitrate of silver, the ulcer should be covered with a piece of fine soft lint, spread with some simple ointment, over which may be placed a bread poultice, of fine linen moistened in the liq. plumbi diacet. dilut., and the whole covered with a piece of oiled silk, or Liston's isinglass plaster." (Parker, pp. 84-5.)

During the two or three days which are generally spent in the application of the caustic, the patient should be prepared by rest, regularity in his living, and mild aperients for any subsequent constitutional treatment that may be thought necessary. Mr. B. Bell has condemned the use of the nitrate of silver to primary sores, under the idea that it produced bubo, and Cullerier still supports this view. Mr. Carmichael, however, proves that this is not the case; and Sir G. Ballingall, after noticing Mr. B. Bell's statements, goes on to say "that of late years he has been in the habit of at once destroying the surface of primary sores with caustic, when of a limited extent, and this practice has been for the most part highly satisfactory; nor," continues Sir G. Ballingall, "could I be justified in saying that it has produced buboes in anything like that proportion which Mr. B. Bell's statements would lead us to expect." As local applications generally to primary sores, Sir G. Ballingall recommends anodyne fomentations, or cataplasms, or stimulating lotions, according to the character of the ulcer; of the latter he mentions particularly the diluted nitric or muriatic acids, solutions of the oxymuriate of mercury, or of the chlorurets of lime and soda.

All irritating or mercurial dressings are to be avoided during the ulcerating stages of chancre, and during the period that the sores are irritable or inflamed. Dr. Wallace states that the most appalling forms of syphilis which he has met with have been owing to the injudicious application of mercurial dressings to chancres in a state of ulceration, irritability, or inflammation. Mr. Carmichael's views correspond.

"Attacks of inflammation........caused by neglect, imprudence, irritating applications, or the use of mercury, may so modify and alter the natural appearance of these primary ulcers, as to render it difficult to ascertain to which class they belong. Under these circumstances, instead of flying to the use of mercury, as is too frequently the custom, I would strongly recommend you to avoid the inextricable embarrassments and difficulties to which this rash step may lead you, and to be contented with directing antiplogistic measures to the necessary extent, mild soothing applications, and rest in the recumbent position, until all inflammation is removed; and then, and not till then, can you be competent to form any correct judgment respecting the true nature of the ulcer, and its appropriate treatment." (Carmichael, loc. cit., p. 253.)

Mr. Key thinks mercurial washes in the early stages of chancre are peculiarly noxious. The morbid action is rather increased than allayed by them, secretion being rendered more copious, and the ulceration more inclined to spread. Ricord condemns the use of ointments generally in the ulcerating stages of chancre; some remedies are employed by him, Cullerier, Desruelles, &c. in the French hospitals, which are found highly useful in the earlier stages of chancre; amongst these may be mentioned the opiate cerate, and the aromatic wine with tannin or opium. The opiate cerate is composed of an ounce of the vinum opii to a pound of adeps, and is very useful as a simple dressing to irritable primary simple sores; a strong decoction of poppy, to which may be added some of the extract of opium, in the proportion of eight grains to the half pint, may also be employed. Ricord's favorite dressing is the aromatic wine of the

French Codex; this is made by digesting four ounces of aromatic herbs (rosemary, rue, &c. &c.) in two pints of red wine for eight days. In certain circumstances, two scruples of pure tannin are added to eight ounces of the wine; and when a sedative application is wished for, half a drachm of the purified extract of opium. The patient is directed to wash the ulceration with these applications gently, without making them bleed, and afterwards to cover the surface of the sore with some fine soft lint moistened with the wine, without making it too wet. To these local remedies M. Ricord attributes the effect of hastening cicatrization, of diminishing the secretion of pus from the surface of the sore, and by their astringent properties, acting upon the surrounding tissues, of preventing the extension of the disease and the formation of fresh chancres, a circumstance very common with the ordinary modes of dressing.

The more mildly primary sores are treated locally, the less likely are they to be followed by those appalling complications which sometimes accompany them, such as rapid ulceration, sloughing, and disorganization of a great portion of the penis and scrotum, and which used to be so common under the old treatment of stimulating mercurial applications during the first days of chancres. We dwell upon this point because we deem it of the first importance: whilst we have almost the universal testimony of modern writers on syphilis in its favour, there are yet some surgeons of the present day in the very teeth of evidence the most convincing, as a glance upon the publications on our table would show, who adopt a sort of routine black and yellow wash system to primary sores of whatever character. To well understand the principles on which the local as well as the general treatment of primary sores must be conducted, the surgeon must constantly bear in mind the two phases of chancre, as established by Ricord and Dr. Wallace, and admitted by Mr. Carmichael and Mr. Key, and most modern writers. In the first stage we have to do with a specific sore, irritable, and poisoned, and poisonous, liable to be irritated by the least stimulus, whilst in the second we have a simple ulcer, destitute of all these characters.

"To the premature use of mercurial dressings, much of the troublesome career of primary sores may be attributed; their injurious action is seen in the conversion of the surface into a yellowish mass, a change that usually indicates ulceration, an increase of secretion, a disposition to spread, and an increased degree of sensibility; and as soon as these applications are replaced by astringents, the changes in the appearance of the sore show on what its previous condition depended. The same remark will not have escaped the experienced surgeon, in the influence of mercury, internally administered, on these sores; and while they remain in the ulcerative stage, it should be sparingly given and cautiously watched." (Key, Guy's Hospital Reports. Oct. 1839, pp. 423-4.)

There are some forms of primary sores to which Mr. Key recommends mercurial dressings in the ulcerating stage. These he employs not as a general rule but as the exception, in sores indolent not sensitive, and secreting but sparingly. Ricord also alludes to this species of primary ulcer, which he describes as indolent and stationary, and in which the secretion is dried up; in these circumstances the aromatic wine, with this exception almost universally employed by him as a general dressing, is to be abstained from, and the opiate cerate or emollient fomentations employed in its stead. We have been in the habit of treating this species of primary sore, which is frequently met with at the orifice of the urethra, by washing its surface with a weak solution of the chloride of lime, and then dressing it with some detergent ointment.

The indurated primary sore demands a treatment both local and general, in some measure different from the ordinary forms of the disease. This, "the classic Hunterian chancre" of Ricord, the indurated primary syphilis of Dr. Wallace, is the form of primary disease for which every author on our list recommends a full mercurial treatment till the sore has healed without induration of the cicatrix. In our account of this species of ulcer, it must be recollected that we limit the expression "indurated" to an ulcer that is so from the commencement of the disease, and do not apply it to those sores which have become indurated from the repeated application of stimulating dressings.

This induration, the natural character of the sore being changed by irritating applications, Mr. Carmichael calls "setting a sore astray," from its regular natural history; and with respect to the particular kind of sore of which we are now speaking it is very liable to be so changed, hence Dr. Wallace says, "when the induration is not the consequence of common irritation from mismanagement." This point is also particularly insisted on by Desruelles. Local treatment is not generally so efficacious in ameliorating the condition of this as the other varieties of primary syphilis, the sore is not commonly much benefited except through the medium of the constitution. Ricord recommends as a dressing to the simple indurated chancre a pommade of calomel and opium. Dr. Wallace also countenances this under the regulations laid down for the treatment of primary sores generally. If suppuration is abundant, the sore is to be washed previously to the dressing with the aromatic wine; should the sore be irritable or inflamed and the ulceration disposed to spread, aqueous solutions of opium, anodyne fomentations, &c. must be used. The application of the nitrate of silver is not so beneficial in this as the other forms of disease, nevertheless both Ricord and Wallace advise its use. The former says "it modifies favorably the surface, and frequently arrests the spread of ulceration;" after the ulcerating stage has passed great advantage will be derived from the gentle application of the sulphate of copper. In the more decided chancre, i. e. the indurated sore of which we are speaking, Mr. Key thinks the "disposition to be set astray is less, and the syphilitic characters being more developed mercurial application agrees better with them, and may be applied with less apprehension of the ulceration extending. In these sores local mercurial action does not render the secretion copious; nor does it render the surface yellow, loose, and spongy, or the edge disposed to break up ......On the contrary, the edge becomes less raised and firm, but not disposed to extend by ulceration; the secretion is altered but not increased; the surface becomes more solid and fibrinous, and inclined to granulate." (Loc. cit., p. 424.)

Dr. Wallace gives some good diagnostic marks by which we are enabled to distinguish between the induration which is the natural attendant upon this species of sores and that which is produced in ordinary simple sores by the injudicious application of remedies. "We may always distinguish by the history of the case and by the character of the areola such indurated ulcerations as are connected with irritation or inflammation. Thus, if indurated primary syphilis be not attended by these morbid states it will not be surrounded by an inflamed but by a callous or livid white areola, with or without a whitish line at the very edge or margin of the ulcerated surface; or else the skin surrounding the ulcer will present its natural appearance." (pp. 310-11.)

There is one symptom succeeding to indurated primary chancres, which sometimes occasions considerable anxiety to the patient and annoyance to his surgeon; this is "induration of the cicatrix." After the healing of a primary sore of this character the cicatrix remains hard and elevated, and is prone to ulcerate anew on the occurrence of the slightest exciting cause. Delpech, Wallace, Ricord, and Cullerier particularly allude to this induration of the cicatrix, which is considered to denote the persistence of syphilitic action in the system, and the "forerunner of accidents to come." It has been proposed to destroy the induration with caustic or to excise it with the knife: both these methods are objectionable; the former frequently occasions foul and untractable sores, which Delpech terms "l'état cancéreux," and the removal with the knife commonly produces a new venereal ulcer more intractable than the first, which in healing may leave a fresh induration behind it. This is a sort of "noli me tangere" disease, which we must be very careful how we interfere with, at least with irritating topical remedies, as escharotics, blisters, or the knife. We seem to have no alternative but to follow the practice of Dr. Wallace, which is at best but a palliative one. "My conduct in this point of practice is to persevere with the use of mercury, provided it agree with the system, for at least ten days or a fortnight after the induration or contraction may have ceased to diminish; and whatever degree of these states may then remain I leave to the slow operation of nature, or to be removed at a future period by the knife, in case this should be desired by the patient, for the purpose of curing aphymosis or any other inconvenient deformity." (p. 313.)

*The Phagedænic Ulcer.* This is the most formidable and uncontrollable variety of primary syphilis. The cause of phagedænic is an interesting matter of enquiry. Desruelles, in the true spirit of Broussaism, attributes it to irritation of the viscera, a chronic gastritis, a gastro-enteritis. Ricord believes also that there is commonly an accompanying visceral irritation, but he believes, moreover, that a cold damp atmosphere disposes primary sores to become phagedænic. Mr. Mayo states "that what gives the phagedænic character to sores on the genitals after infection is some peculiarity of the general habit." This is perhaps true, but the difficulty is to know in what this peculiarity consists. We have sometimes seen inflammation, mortification, and death succeed the slightest scratches; and this is not uncommon in the draymen and porters of London, who consume large quantities of gin and porter.

Phagedæna is generally divided into the ulcerative and sloughing. Dr. Wallace has attempted a classification which he thinks "may be of some practical importance towards the discrimination and management of these frequently formidable diseases."

*Phagedænic Primary Syphilis.*

| 1. *Without Slough.* | 2. *With White Slough.* | 3. *With Black Slough.* |
|---|---|---|
| A. Simple. | A. Simple. | A. Simple. |
| B. Inflamed. | B. Inflamed. | B. Inflamed. |
| C. Irritable. | C. Irritable. | C. Irritable. |

The local remedies best suited to the different varieties of simple phagedæna are the nitrate of silver, either solid or in saturated solution, applied with a pencil; the pure nitric or nitro-muriatic acids, the white muriate of antimony, or a saturated solution of the oxymuriate of mercury in alcohol. In the sloughing or foul varieties of the simple phagedæna, Ricord recommends as very serviceable alternate dressings of the aromatic wine and a detergent ointment: the surface of these sores may be washed frequently with strong solutions of the chlorurets of soda and lime.

In the inflamed variety we must employ rest, "general and local" bloodletting, emollient fomentations or lotions, and poultices, purgatives, antimonials, and abstinence. Ricord says he is very wary of local bloodletting in this or any other form of primary syphilitic sore. He condemns the practice so common in many of the French hospitals of applying leeches in the centre of the sore, which commonly occasions an extension of the ulcer in depth to the extent which has been divided by the bite of the leeches; there is also a paramount objection to their application in the immediate vicinity, which is the inoculation of the leech-bites by the secretion from the surface of the ulcer, which is most likely to occur, and the consequent formation of fresh venereal sores.

In the inflamed varieties of phagedæna with white or black slough Dr. Wallace thinks the application of the nitrate of silver hurtful, and in fact we should do well to observe what he says of its use in phagedæna generally, that used too long or repeated too often it will produce consequences in some respects as unpleasant as those which result from an overdose of mercury. In the inflamed phagedæna with or without slough a purely antiphlogistic treatment, with anodyne fomentations and poultices, is the safest practice; leaving the application of stimulants and caustics till all local and constitutional irritation has subsided.

The irritable phagedænic ulcer demands a local treatment different from that of either of the before-mentioned varieties. Ricord and Wallace employ the nitrate of silver, the liquor arsenicalis, or the strong nitrous acid. These, however, frequently dispose the ulceration to spread, particularly where the sore is surrounded by a diffused areola. When this is the case the opiate cerate or strong aqueous solution of opium may be employed, whilst this remedy should be given freely at the same time by the mouth. Amongst preparations of this character Dr. Wallace speaks highly of mercurial ointment with the ext. opii, a drachm of the latter to an ounce of the former, or the mel rosæ with laudanum. As a general application to these sores, in our own practice, we have succeeded better with turnip or carrot poultices than with almost any other form of remedy. There are, however, individual circumstances depending on the character of the sore, its degree of indolence, irritability, or inflammation, in which the remedies we have mentioned will find their application.

The writers before us are divided as to the propriety of the mercurial treatment in phagedænic primary syphilis, Mr. Carmichael agreeing with the French school in rejecting the remedy as most dangerous and deceptive in every variety of the disease. "The miserable mutilations and sufferings," he says, "which our soldiers endured in Portugal from phagedænic and sloughing ulcers, at the commencement of the Peninsular war, by the exhibition of mercury, might have inspired the deputy-inspector of hospitals with a desire to obtain information on the treatment of venereal complaints from every source; for our army surgeons soon discovered that the *black lion* of Portugal, as the sloughing ulcer was termed, could not be tamed by mercury, and that without giving a grain of it the Portuguese practitioners knew better how to effect their object." Sir G. Ballingall, after an experience of many years, informs us in his recent work that he quite accords with Mr. Carmichael. Mr. Key, in his report of the primary syphilitic cases occurring at Guy's hospital, states that in the constitutional treatment of these sores mercury is wholly inadmissible. "It tends," he says, "to increase irritability, to lower the powers of the patient, and therefore to quicken the phagedænic action. Loss of rest, and the irritability of the arterial and nervous systems to which it gives rise, are the prominent points in these cases." We perfectly agree with these authors in condemning the use of mercury generally in phagedænic primary syphilis, yet we are of opinion, and the results of every day's experience confirm this opinion, that there are cases of this character frequently occurring in which mercury judiciously employed is our best remedy; and this is just the way in which Ricord views the question, when, in speaking of phagedænic chancres, he says (p. 573) "there are circumstances under which a mercurial treatment is followed by the best results, and this fact is constantly proved by the practice of those who avow the most deadly hatred to mercury." It remains, however, to be determined what are the forms of phagedæna in which mercury may be employed with advantage. Ricord thinks it impossible in the present state of science to point out with certainty what these circumstances are. If the disease continues to advance in spite of the usual remedies judiciously employed, as a sort of forlorn hope he has recourse to mercury, first in form of local application and subsequently by the mouth or friction. He is regulated in the continuance of the remedy by the effects produced, continuing it if the disease appears inclined to yield, or giving it up should it still continue to extend. Mr. Lawrence, whilst he condemns the indiscriminate use of mercury in phagedæna, believes there are circumstances in which it may be employed with advantage, though he, like Ricord, does not seem able to point out what these circumstances are. He states that it has often happened to him to see cinnabar fumigations employed as a local remedy in phagedænic ulcerations of the throat accidentally cause copious salivation, and that in many instances where he had seen this he had found that the local disease in the throat, as well as in other parts, proceeded very favorably; so that he would not lay it down as an absolute rule that mercury ought never to be employed in these cases in reference to its general effect upon the system.

We think Mr. Carmichael has written in too exclusive a spirit of partisanship when he condemns mercury as poisonous in every variety and form of phagedæna, particularly when he goes so far as to say that he would not even permit the use of a few grains of cinnabar as a fumigation, for fear of mercurializing the whole system. The recorded experience of most modern writers on syphilis, to whatever school they may belong, proves that there are constantly occurring cases of phagedæna so intractable under the ordinary treatment that mercury is fled to as a last resource, and there are many of these in which it is perfectly successful. "Nor must it be denied," says Mr. Mayo, "that occasionally a short and brisk course of mercury will give a new turn to the complaint and cause these troublesome sores at once to close. This remedy, however, should be last resorted to." (p. 20.)

Dr. Wallace has endeavoured to discriminate between those forms of phagedæna in which mercury should be used and those in which it should be abstained from : he believes that all the varieties of phagedænic primary syphilis have hitherto been very much confounded together, to the injury of the patient and the confusion of the practitioner. Dr. Wallace's classification of these sores is not founded on the incipient or primary character of the sore, but upon the appearances it may assume during its course, which evidently depend on constitutional causes; the chief divisions being founded upon the degree of irritability or inflammation attending a primary sore, or the character of the slough with which it may be covered; this will be seen by referring to Dr. Wallace's table already quoted. In the first variety of phagedæna, the simple primary sore without slough, characterized "merely by an unusual persistence or activity of the specific action attendant on the ulcerative stage of primary syphilis," Dr. Wallace thinks mercury almost indispensable, and recommends the patient to be brought as quickly as possible under its full influence. "But," he adds, "if the patient has been dabbling with mercurial remedies, and if there be reason to suppose that his constitution has been in consequence more or less disordered, we shall act more judiciously by suspending for a time the use of mercury, and endeavour by proper measures, but principally by attention to the mode of living of our patient and by the use of the mineral acids with sarsaparilla, to restore the system to a state of tranquillity before we enter again on mercurial treatment; which may, however, be then used with success." (p. 137.)

In the inflamed variety of simple phagedæna the first indications are the reduction of the inflammation according to the principles already laid down. Mercury may be used at the discretion of the practitioner, when the ulcer has put on the reparative process, in the way before recommended for simple primary sores.

In the irritable variety of simple phagedæna without slough, mercury is to be interdicted. Dr. Wallace believes that in this form of the disease the remedy is always injurious, and if persevered in produces an increase "of irritability local and general, and hectic and secondary symptoms of a very unmanageable kind may result." In this form conium, hyoscyamus, opium, and Dover's powder, but more particularly opium, locally and generally, are the chief remedies to be relied on, with sarsaparilla in conjunction with the iodide of potassium ,or the mineral acids, or Mr. Carmichael's favorite remedy, the cold compound or simple infusion of sarsaparilla in lime water. It is in these cases that Mr. Lawrence also thinks sarsaparilla most beneficial. In the three forms of phagedænic syphilis without slough, Dr. Wallace recommends a full course of mercury in the first or simple form ; an active antiphlogistic treatment to precede the mercurial treatment in the second or inflamed variety; and measures calculated to tranquillize the system in the third or irritable.

In the "simple phagedænic primary syphilis with white slough," Dr. Wallace recommends mercury to be freely exhibited, "wherever it may be seated and of whatever extent it may be." With the constitutions, however, in which this sore occurs mercury is very apt to disagree; and although the disease may be influenced beneficially and the remedy properly administered may quickly excite such sores to separate their sloughs and cicatrize, a prolonged use of the remedy commonly induces a state which Dr. Wallace calls "febrile mercurial irritation:" and if mercury be continued after this condition of the system has been induced the sore soon becomes "stationary and sensitive," and afterwards "painful and spreading," whilst secondary symptoms of a malignant character frequently set in.

This species of disease is particularly liable to occur at the orifice of the urethra, or just within the opening of the glans; and we have seen cases characterized to the patient on the onset merely by a sanious discharge from the urethra where this passage has been speedily perforated and the glans partially or totally lost. We would impress upon the surgeon the necessity of examining the urethra in all discharges from it of this kind, since in many instances, two of which are now under our own care, these discharges are from the surface of the white phagedænic chancre seated just within the orifice of the urethra. Dr. Wallace alludes to cases of this kind, p. 157.

The characters of the "inflamed phagedænic primary sore with white slough," are a slough of white colour at the junction of the sore with the living parts, which parts are in a state of inflammation. The distinctive character of the sore is to be drawn from the colour of the slough at the junction of the ulcerative with the living parts, and not from the colour of the slough on other parts of the sore, since these occasionally become black or green from the exposure of the surface to the atmosphere. The first indication in the treatment of this sore is the reduction of the inflammation by an active antiphlogistic treatment, employed, according to the circumstances of the case, generally and locally. We would beg, however, the attention of the reader more particularly to the point we are now endeavouring to make more clear; viz. the indications for the use of mercury in the different forms of phagedæna; and with respect to the sore under notice Dr. Wallace states that he has ascertained "an important fact,—that mercury, if given in full doses, or so as to produce rapidly its effects on the system, will control, even in its most inflamed state, the progress of the phagedænic disease just described." (p. 168.)

Dr. Wallace "does not mean to say," that this practice should in all cases be ordinarily adopted, but believes that in many cases it will afford the chief if not the only protection to the patient when the disease is making havoc amongst parts of great importance. When employed in this form of disease it must be combined with an antiphlogistic general treatment, as general and local bleeding, and nauseating doses of antimonials. In support of this position Dr. Wallace adduces a case of obstinate and destructive inflammatory phagedæna. In this instance "much time had been spent in fruitless endeavours to control the progress of the disease," the usual remedies had been tried in vain, till the prepuce was lost, with part of the glans, and a large portion of the covering of the testicles. So great was the attendant irritability and inflammation that mercury "was the last remedy one would have ventured to try." The patient was importunate, and threatened to leave the hospital if mercury were not used. Large alterative doses had been employed without effect; it was now given in full doses: "his mouth was made very sore, and the progress of the disease was not only controlled but almost entirely stopped." Dr. Wallace noticed that "wherever the disease had been extending by a white slough, there or on such parts, though inflamed, the mercurial treatment had acted most beneficially; but that, on the contrary, at some points, where the sore was extending by a black slough, its progress remained uncontrolled or rather aggravated." (p. 170.) Dr. Wallace is not satisfied with the degree of inflammation surrounding a primary sore as a rule for the non-employment of mercury, and founds his opinion upon the value of mercury freely administered in syphilitic iritis, and upon the fact that mercurial fumigations, in certain destructive sores of the throat, are frequently very beneficial, although attended apparently by great inflammatory action. The result of Dr. Wallace's experience in the employment of mercury in these cases has led him to establish a rule to which we are disposed to attach very considerable importance: "That although that form of inflammation which supervenes when a patient is under a mercurial course is sure to be aggravated by persisting in the use of mercury, this remedy will powerfully assist to subdue inflammation which commenced under different circumstances." (p. 175.)

In the "irritable variety of phagedæna with white slough," Dr. Wallace also thinks a short but full course of mercury the best remedy, if the case have not been previously mismanaged. When it is used it must be very carefully watched, and combined with large doses of opium, Dover's powder, cicuta, hyoscyamus, sarsaparilla and the carb. of ammonia, whilst the local treatment must be anodyne and unstimulating. All the forms of phagedæna with white slough are, in the opinion of Dr. Wallace, favorably influenced by mercury. He considers this form of disease to be the result of inordinate action, "syphilis in an acute form, modified by peculiar states of constitution." These peculiar states of constitution, of whatever nature they may be, must render the surgeon circumspect in the employment of mercury, particularly in the outset of the disease, or as a specific remedy; but where the ulceration spreads with rapidity, uninfluenced by the general treatment, and threatens the loss of important parts, mercury must be used; but, says Dr. Wallace, "until our path be more clear we shall run much less risk, in doubtful cases, of doing mischief by refraining from than by employing mercury." (p. 179.)

The "simple primary phagedæna with black slough" of Dr. Wallace is analogous to the sloughing ulcer of M. Bacot, and is evidently merely a variety of the true Hunterian chancre, an indurated primary sore with a dark or livid coloured slough. Its characters are "much greater induration of the surrounding parts than any of the other forms of primary phagedæna, scarcely any inflammation, and attended by very little sensibility." Dr. Wallace states that it requires directly any other form of treament than the regular primary disease. It is often very much benefited by the internal employment of the nitrous or nitro-muriatic acids; but "the actions of reparation and perfect cicatrization may be produced in it with much more certainty, in a much shorter time, and with less expense to the constitution, by a mild and well-regulated course of mercury than by any other means." (p. 185.)

The disease, however, generally occurs in constitutions very susceptible of the mercurial influence, and hence the exhibition of the remedy requires more caution in its management than in the simple primary sores. Dr. Wallace thinks that great advantage will be derived from the occasional interruption of the medicine and from substituting during its omission remedies that are calculated to improve the general health and tranquillize the nervous system.

The two remaining forms of phagedæna are the inflamed irritable varieties with black slough, the sloughing phagedæna of authors generally, the gangrenous ulcer of Mr. Bacot, and the "chancre phagédenique gangreneux" of Ricord. In these forms all writers agree in condemning the use of mercury; the disease is to be treated on the principles which should regulate us in the management of similar diseases arising from causes not syphilitic.

It must be recollected, however, when the sore has been brought to granulate and heal by proper remedies, that it has had a venereal origin, and perhaps has succeeded to a regular primary sore, rendered gangrenous by irregularities and bad management, and that it may be followed by secondary symptoms. "It is more than probable," says Mr. Bacot, "that secondary symptoms will not ensue, and if they should it will be time enough to arrest them when they appear. But it is, I think, not only fair towards the patient but prudent to explain to him the probabilities of the case, that his attention be awakened by any deviation from his usual state of health, and that no time be lost under a mistaken view of his condition."

In concluding these notices of the employment of mercury in different forms of primary syphilis, we beg impressively to call the attention of our younger readers to Boerhaave's remark "that he knew of no remedy but what became so by proper employment," and to that of Sir G. Ballingall, that "in the hands of a judicious practitioner mercury will fulfil a number of different and apparently contradictory indications; in the hands of ignorance, temerity, or indiscretion it will do more harm than good." (p. 438.) It certainly must be used in a truly eclectic spirit, and not in conformity with the exclusive spirit of partisanship, or the dogmas of this or that school.

The work of Lucas Championnière, which contains an account of the views of Cullerier, and the practice adopted by him at the Civil Venereal Hospital, Paris, has some valuable remarks on the pathology and treatment of chancres in the female, and with a notice of these we shall conclude our account of the views of modern authors on "primary syphilis." M. L. Championnière begins by remarking that all that has been hitherto written on the contagion of syphilis before the discovery of the speculum and its application to the examination of the deep-seated portion of the vagina and the neck of the uterus is to be considered as perfectly valueless, since the disease in the female which contaminates the male is much more frequently seated on the neck of the uterus than on the external parts. There are constantly presenting themselves at the venereal hospital females who have no trace of disease on the external parts, but who apply for assistance because they have produced disease in healthy men who have cohabited with them. On examination with the speculum these females are found to suffer from different forms of disease of the neck of the womb which is not manifested by any external sign, or in fact by any symptom whatever. These facts noticed by M. Cullerier corroborate Dr. Ferguson's oft-told tale of the Portuguese opera-dancer. The affection of the neck of the womb presents numerous forms, all of which M. Cullerier thinks are capable of producing ulcers of the penis from cohabitation. In the first form the neck of the uterus is red, swollen, and hypertrophied, but not in a sate of ulceration. If erosions exist, says M. Cullerier, they are so slight as not to be perceived by the naked eye. The state of the neck of the womb in this variety of complaint, resembles very much that of the glans penis in balanitis or external gonorrhœa. More frequently the neck is covered with ulcerations, more or less numerous and more or less deep, from which oozes a virulent sanies. M. Cullerier thinks these are true chancres. They resemble chancres of the penis, in their history, their duration, and more particularly in the fluid they secrete. Occasionally M. Cullerier has found the true indurated chancre upon the neck of the womb, though this form is not so commonly met with as the other varieties. We shall not follow M. Cullerier through the treatment he recommends for primary sores in this situation. They are to be dealt with in the same manner as primary venereal sores generally, the situation in which they occur is the only circumstance which can in any respect modify the treatment, and hence it is almost impossible to cure them properly without the occasional use of the speculum to separate the parts for the purpose of cleaning them thoroughly by injection, or applying any local remedies the nature of the sores may require. M. Cullerier states that the situation in which these sores occur renders their speedy cure very difficult, and whilst chancres on the penis are generally healed in two or three weeks, these diseases of the neck of the womb commonly require as many months. M. Desruelles corroborates also Cullerier's statements of the frequent existence of true venereal sores on the neck of the uterus. In the deep-seated parts of the vagina chancres are rare. These chancres may exist as we have before shown an indefinite period of time without producing constitutional symptoms, and without the patient being aware of their existence.

In the excellent memoir of Dr. Hauck, most of the conclusions of the French authors relating to the primary syphilitic affections in the female are corroborated by the results of his own experience; and the absolute necessity of the speculum in the diagnosis and treatment of these cases is clearly demonstrated.

II. Constitutional Syphilis. The phenomena of constitutional syphilis are divided by M. Ricord into secondary and tertiary, a division which in some measure harmonizes with the ideas of Hunter developed in his account of the first and second order of parts affected in lues venerea. M. Ricord thinks that the reason why the treatment of this disease is commonly so uncertain and unsatisfactory is because its natural history is not clearly understood; hence the necessity of dividing syphilis into different stages, all of which are perfectly distinct from each other in their pathology, their phenomena, and the parts of the body in which they make their appearance. A distinct treatment is, in M. Ricord's view, to be framed for the management of each of these varieties, the remedies which are useful in one stage being comparatively inert in the others.

Primary syphilis may be succeeded by a series of affections which are termed by Ricord "successive," but are not truly secondary. Such are the diseases which result from the simple extension of the primary local disease, and of this character are fresh chancres, abscesses simply inflammatory or virulent, and buboes partaking of the like distinction.

Secondary symptoms, or those of general infection, arise when the venereal virus has undergone some degree of modification by being mixed with the blood and thus producing what M. Ricord terms the "syphilitic temperament." The diseases which may properly be termed secondary seldom occur before the end of the second week after the appearance of the primary disease, more commonly, however, they appear about the fourth, sixth, or eighth week, and commonly at a much later period than this. Secondary syphilis attacks the skin, the mucous membranes, the eyes, the testicles, &c. The great peculiarities to be observed in these forms of the disease are that they are not capable of being transmitted by inoculation, but from parent to child by hereditary taint. These "secondary accidents" of Ricord correspond to Hunter's first order of parts affected in "lues venerea," which he states to be the skin, tonsils, throat, inside of the mouth, and sometimes the tongue.

Tertiary symptoms appear at an indeterminate period, succeeding the primary disease, but commonly not till a long period after this has been cured, and in a majority of instances not till after secondary symptoms have appeared, or whilst they are still present. The tertiary symptoms are nodes, tubercles of the skin or cellular tissue, periostoses, exostoses, diseases of the bones and fibrous system generally, and many internal affections not clearly defined. The tertiary symptoms are neither capable of propagation by inoculation nor of transmission by hereditary taint —at least under any specific form of venereal affection. The tertiary symptoms are analogous to Hunter's affections of the second order of parts, which consists of the periosteum, fasciæ, and bones. It is in this latter class of affections that M. Ricord thinks the iodide of potassium so worthy of confidence.

Before entering into the consideration of the treatment, properly so called, of the constitutional forms of syphilis, M. Ricord devotes a short chapter to the examination of the question of the preventive treatment, remarking that it is against this that our care should chiefly be directed, since the primitive diseases are comparatively easy of cure by various remedies, and sometimes disappear spontaneously.

M. Ricord believes the probability of the occurrence of secondary symptoms to be in some measure in proportion to the time that the primary sore remains open. He has never seen a case in which the primary sore healed in five days has been followed by secondary symptoms; hence, says M. Ricord, the best rule that can be given for the prevention of secondary symptoms is the radical cure of the primary disease, without induration of the cicatrix, in the shortest space of time possible. And for this purpose he speaks with more confidence of mercury than of any other remedy, but considers it valuable rather as a remedy calculated to cure the primary disease than to prevent the secondary.

If there is any kind of treatment calculated more than another to favour the appearance of secondary symptoms it is that which has not radically cured the primary disease. M. Ricord thinks that all persons are not susceptible of secondary symptoms, and he states that the occurrence of the latter is favoured by peculiar idiosyncracies, not well defined. Sudden changes in the accustomed mode of living of the patient or his habits generally, pregnancy, the critical period of life, gastric disturbance, habitual irritation of the throat, mouth or skin, scrofula, &c., M. Ricord specifies as causes predisposing to the occurrence of secondary affections: hence, in addition to the radical cure of the primary symptoms, our second grand principle is attention to the general health.

It will be quite unnecessary to follow M. Ricord through his account of the general treatment of secondary syphilis. The general remedies are included under the heads of antiphlogistics, diet, baths, the state of the primæ viæ, and some other points of minor importance. In reference to these heads, as to most others, M. Ricord is purely eclectic in his practice. Attached to no particular school or dogmas, he states merely what remedies have succeeded best in the vast number of patients he is called daily to treat. Apart from any specific treatment in the management of secondary syphilis, he adopts that general system of hygiène which agrees best with his patient. We agree with him, however, in thinking that no specific rules can be laid down; they must be left to the discretion and judgment of the surgeon.

It is to a mercurial treatment in confirmed constitutional syphilis, says M. Ricord, that we must give the preference, provided there are no contra-indications. The deductions made by M. Humbert from the results of M. Biett's practice at the hospital St. Louis are,—That of all remedies mercury is the most efficacious in curing constitutional syphilis which exists without acute inflammation, but that it is better abstained from in irritable subjects feebly constituted. It cannot be denied, continues M. Humbert, that the preparations of mercury cure confirmed secondary syphilis more frequently than any other remedy. Mr. Mayo says, "of the remedies for constitutional syphilis, mercury is so far the most important that it is the only one, however rarely it may be necessary to use it with this object, through which the immediate extinction of constitutional lues can be anticipated; and that besides as a repellant of the disease, it is one of the most efficacious." (p. 61.)

The preparation of mercury chiefly employed by M. Ricord in the treatment of constitutional lues is the proto-ioduret, the hydrargyri iodidum of the London pharmacopœia. He commences with one grain for a dose, combined with opium, and carries it as far as six in the day, but does not exceed this quantity. Cullerier prefers the iodide in the constitutional forms of the disease, administering it, at the venereal hospital, in the proportions of half a grain night and morning, combined with opium and guaiacum. In Biett's hospital, the St. Louis, the bichloride is generally preferred in very small doses, and this was Dupuytren's favorite remedy.

A certain degree of restlessness or irritability generally accompanies constitutional syphilis, which is so much benefited by opiates that M. Biett's practice has constantly furnished numerous cases where these affections have disappeared under the influence of opium alone, uncombined with mercury. The same views are supported by Ricord. Cullerier states "that opiates possess very marked anti-syphilitic properties, and even administered alone have produced some very remarkable cures." The term anti-syphilitic is clearly not to be understood in this sentence in a specific sense, but there can be no question of the vast utility of opium, used both generally and locally, in all forms of the venereal disease. Dupuytren was in the habit of using the bichloride combined with the extract of aconite with the same view.

Wallace, Ricord, Cullerier, and most other surgeons have lately employed the iodide of potassium largely in the various forms of constitutional syphilis. Ricord (Gazette des Hôpitaux, March, 1839,) has established that its results are most happy in the tertiary forms of disease, in affections of "Mr. Hunter's second order of parts," in which he considers it the remedy "par excellence." Iodine of itself is a powerful anti-syphilitic, but, unless in a state of combination with mercury, is inadmissible in the treatment of the simple primary forms of disease. The iodide of potassium is chiefly employed by Cullerier in chronic syphilitic affections of the testicles, and of the inguinal and cervical glands, also in cutaneous affections and diseases of the fibrous or osseous systems which are of syphilitic origin. M. Cullerier thinks the effects of the iodide of potassium are less prompt than those of mercury, and hence when the stomach bears it well it requires a longer employ. He exhibits one grain of iodine with from two to four of the iodide of potassium dissolved in one ounce of water, and given at intervals in any convenient vehicle during the day. He does not increase the quantity of iodine beyond two grains in the day, nor that of the iodide of potassium beyond ten.

The most complete account of the mode of administration of the iodide of potassium and its effect on different forms of constitutional syphilis is that given, from the results and experiments made by Ricord, in the Gazette des Hôpitaux, for March 19, 1839. M. Ricord employs the iodide of potassium in gradually increasing doses, commencing with ten grains dissolved in three ounces of distilled water, and given at intervals during the day in any convenient vehicle. This quantity may be increased or diminished according to the effects produced; when the remedy agrees, which it most commonly does if the stomach be healthy, the dose is increased ten grains every two or three days till it is carried to a drachm, a drachm and a half, or even more, in the course of the day. The iodide of potassium, in full doses, occasions a sensation of warmth in the stomach, improves the appetite, accelerates digestion, and quickens the pulse; a common and almost constant effect is a great increase in the secretion of urine. Sometimes it produces a ringing in the ears, a slight pustular eruption or diarrhœa. When these occur the dose must be diminished or the remedy abandoned altogether for a few days, till these symptoms have passed away: its use is then to be recommenced in smaller doses than before. In the great number of patients who have been treated by M. Ricord, the beneficial effects of the iodide of potassium have been constant but not produced with equal rapidity. In this respect there has been considerable variation. We do not agree with Mr. Mayo that "no medicine, where it does good, produces amendment so speedily as the iodide of potassium."

M. Ricord mentions the syphilitic deep-seated tubercle as the first disease in which he has extensively tried the iodide with success. The original seat of these tubercles, Mr. Babington says, is in some structure below the surface of the cutis, probably in the sebaceous glands. Ricord speaks merely of this disease as deep-seated tubercle; it belongs to his third order of parts, is prone to inflame and ulcerate, and terminate in the formation of foul, spreading, destructive sores. Before the tubercles have become inflamed or softened, whilst they are still indurated, M. Ricord has constantly succeeded in dispersing them by means of the iodide of potassium with small doses of the iodide of mercury. He employs the iodide of mercury in one grain doses, with three, four, or five of the ext. conii, and the iodide of potassium in gradually increasing doses. The mercury is rarely continued for a long period, and very commonly M. Ricord makes use of the iodide of potassium alone. When the tubercles have become ulcerated M. Ricord still relies upon the iodide of potassium as his principal remedy. The local treatment, however, is of great importance, and must be directed under the views which would regulate us in the management of primary venereal sores.

M. Ricord thinks that mercury is generally inefficacious in the tertiary forms of syphilis. M. Biett has found in his extensive opportunities for observation, that mercury did not produce any marked effect over the syphilitic tubercula. He mentions some cases treated successfully by the arseniate of soda. Mr. Mayo alludes to a case in which mercury was given without any benefit. (p. 88.) Mr. Parker's work contains an account of the local remedies to be employed in the ulcerating forms of the "tubercula." (pp. 141-6.) From the extensive trials made by Ricord, no remedy appears to have been so generally useful as the iodide of potassium.

The tubercular forms of syphilis commonly occur in conjunction with very considerable disorder of the general health, and it is scarcely necessary to notice again the stress laid by all the authors before us on the propriety and indeed absolute necessity of attending to this before any specific treatment is adopted. On this point M. Ricord has some admirable remarks, (p. 615.) When the secondary forms of constitutional syphilis occur without complication in a good constitution not previously impaired by other diseases or weakened by improper treatment, a specific treatment may be at once adopted, and the cure is commonly easy and quick. When constitutional syphilis is complicated the complications should never be neglected; if it occur in conjunction with acute affections the treatment of the latter ought to be our first care, with the view of reducing the venereal affection to the simplest form possible. If chronic diseases, as those of the skin or the viscera, are found in connexion with it, the syphilis should first be treated, taking care that the special treatment of the latter be so framed that the concomitant disease, of whatever character it may be, is not aggravated by it. "Exclusive treatments," says M. Ricord, "to whatever doctrine they belong, are the least likely to succeed."

Amongst other forms of disease more particularly benefited by the iodide of potassium, M. Ricord mentions nocturnal pains in the bones, and diseases of the bones and periosteum. With respect to the former, when they have been fugitive they have generally yielded to the iodide: when localized for a longer or shorter period this remedy, in conjunction with blisters over the affected parts, has most commonly succeeded. M. Ricord believes nocturnal pains in the bones to result from a true inflammation of the osseous tissue, from syphilitic taint, and hence he considers these pains as the first symptoms by which such disease is characterized. These pains are commonly attributed to the prolonged or excessive use of mercury, but M. Ricord frequently meets with cases of diseases of the bones and periosteum in various stages at the venereal hospital in patients who have not taken a grain of mercury, and who have had chancre as a regular antecedent of the disease. Here, as in syphilitic caries, M. Ricord relies chiefly upon the iodide of potassium; he believes that mercury is generally injurious, at least he has abandoned its use, and succeeds better with the iodide. The iodide of potassium merits, in M. Ricord's view of its properties, in the treatment of the tertiary forms of syphilis, all the confidence that mercury has obtained in the treatment of the secondary, to which forms M. Ricord considers the iodides of mercury are particularly adapted.

It now only remains for us to notice briefly the merits and tendencies of the several works which have furnished the materials for this article.

The first in place as well as in character is the volume of M. Ricord. M. Ricord is one of the surgeons of the Parisian Civil Venereal Hospital, and the monograph under notice must be considered as the result of the experience acquired by him in this extended field of enquiry. The work is divided into three parts: the first is devoted to the literary history of inoculation in syphilitic diseases given above; the second contains a detailed account of the cases from which the deductions have been made, and also the treatment adopted in each case, so that this part of the work is moreover a clinical report under the heads of chancre, gonorrhœa, buboes, and constitutional diseases; the third is a summary of the treatment which M. Ricord has found most successful at the venereal hospital and in his own private practice. This last division is worthy of the more confidence since it is purely eclectic; M. Ricord having no therapeutical dogmas to support, he is a partisan of no particular school, and both simple and mercurial treatments are adopted by him under the circumstances in which each is the most likely to be followed by success. Many of the points established by the researches of M. Ricord tend to confirm the doctrines which Hunter taught. Of this character are the proofs pertaining to the non-propagation of the secondary forms of syphilis by inoculation. The pathological division of venereal diseases which M. Ricord has proposed, of the primary, secondary, and tertiary forms, seems founded on the original idea of Hunter. On this point, however, M. Ricord has added much to the natural history of the disease; and, what is of perhaps more importance, he has shown that certain remedies are better suited to one stage than others—in other words, that each of the forms has in some measure its separate mode of treatment: here he has distinctly pointed out those varieties of the disease in which the iodide of potassium is most useful, and again others where mercury is most likely to succeed. M. Ricord has first clearly shown that the discharges from the urethra are sometimes indicative of a chancre seated within this canal: this has also been admitted by his colleague Cullerier, and suspected by Dr. Wallace; but the event of having clearly explained the existence and differential diagnosis of the "chancre larvé" is due to M. Ricord. We recommend M. Ricord's work to all interested in the treatment of syphilis, as the best treatise on the disease since the days of Hunter, and in all respects worthy of the confidence of the reader.

The work of M. Lucas Championnière, contains a full account of the practice of M. Cullerier, senior surgeon to the Civil Parisian Venereal Hospital and colleague of M. Ricord. M. Cullerier's practice is also an eclectic one. Although leaning towards the opinions of the non-mercurialists he admits, what most of them deny, the existence of a special venereal virus. The exposition of the practice of M. Cullerier in the work before us is made under two grand sections, the first consisting of general views of the nature of the disease and its treatment, with a *coup d'œil* of the inconveniences of the mercurial treatment; and the second division giving the special indications for the employment of different remedies; amongst these are particularly mentioned mercury, iodine, gold, platina, opium, decoctions of the woods, the carbonate of ammonia, and antimony. These special remedies, the indications for which are pointed out by M. Cullerier and the different forms for their administration given, are to be used under the influence of a general treatment, the chief agents in which are confinement to bed, a low diet, bleeding, aperients, and the warm bath.

We have not space enough to follow M. Cullerier through the various details of his practice. The work contains many valuable observations, and generally it may be said to advocate a mixed or modified mercurial treatment.

As in what has been hitherto noticed in the authors under review, we have not said anything of bubo, we shall take this opportunity of mentioning M. Lucas Championnière's account, given in the volume before us, of the plan adopted in the venereal hospital of treating buboes with blisters and a strong solution of the bichloride of mercury. This plan, originally proposed by M. Malapert, a French army-surgeon, consists in applying a small blister over the centre of the tumour, the next day removing the epidermis which has been raised by the blister, and applying a small compress over the denuded surface, soaked in a solution of the bichloride of mercury in the proportion of twenty grains to the ounce, this compress is kept in place by straps for two hours and is then removed; at this period an eschar involving the skin to a greater or less depth is formed, which on separating leaves the bubo very much diminished in size, and is generally followed by its total disappearance. After the eschar has been formed, the swelling may be covered with a cold lotion or poultice. This plan of dispersing a venereal bubo has now the sanction of very great experience drawn from its successful employment in the French army, and for seven years in the great Civil Venereal Hospital under the direction of Cullerier and Ricord. Cullerier speaks in very positive terms of this remedy from his own observations of its utility, and it remains for us merely to indicate those circumstances in which it is most likely to succeed. It is well known that the sympathetic bubo, succeeding to gonorrhœa, is not so prone to suppurate as that following chancre, and here M. Ricord prefers attempts at dispersion, to be made first by pressure with the ammoniacum and mercury plaster; if these do not quickly accomplish their object, or the tumour tend to increase, the blister with caustic is used. In all forms of the venereal bubo, except where matter has actually formed, or the surface is very much inflamed, it may be employed. Resorted to early in the disease, Cullerier states that he does not recollect seeing it fail in producing a total dispersion of the tumour.

The new edition of Dr. Wallace's work, bearing date 1838, is a mere reprint; but as some opinions there given are defended by him in his lectures in the Lancet in 1836-7, we presume they have undergone no alteration. This volume notices only the primary forms of syphilis, which Dr. Wallace divides into the simple, the phagedænic, the superficial, the catarrhal, the indurated, the annular, and the fungoid primary forms of disease; it also includes bubo. Some valuable practical remarks are to be found scattered all through the volume, particularly on the treatment of simple primary sores. Mercury is too indiscriminately recommended by Dr. Wallace, who employs it in every form of the primary disease, not excepting gonorrhœa (which is Dr. Wallace's catarrhal syphilis), and some forms of irritable or inflamed phagedæna. The practical surgeon, however, may study this volume with much advantage.

Mr. Mayo's little work is not a complete treatise on syphilis, but a reprint, with some additions, of his lectures on this subject which have already appeared in the Medical Gazette. The greater part of the volume is devoted to the constitutional forms of disease. The general plan of treatment recommended is a modified mercurial one. Mr. Mayo has used the iodide of potassium in many cases, the results of which have borne out what modern experience has already made known in reference to the beneficial effects of this remedy.

We noticed Mr. Parker's little work in our last Volume (p. 239); and would not have referred to it here but from the use we have made of its contents in the present article. It is purely eclectic as regards modern theories and practice in syphilitic diseases. Mr. Parker advocates a modified mercurial treatment, but shows at the same time that mercury is most successfully administered in accordance with the tenets of the simple treatment. The first section is devoted to an account of the non-specific treatment of syphilis, constitutional and local; the second treats of the different preparations of mercury and their mode of employment, and the particular indications for their use; the third considers inoculation as a means of diagnosis, and gives a full account of M. Ricord's experiments on this subject. Several new modes of treatment successfully followed in the venereal hospitals of Paris and Berlin are here first introduced to the English surgeon: amongst these may be mentioned Ricord's treatment of chancre by the aromatic wine; Malapert's and Cullerier's plan for dispersing bubo by blisters and the caustic solution of the oxymuriate of mercury, &c.; and Biett's management of the venereal diseases of the skin at the hospital of St. Louis. The work taken as a whole is a compendious digest of modern practice, more particularly as it is adopted by surgeons at the head of the great venereal hospitals in France and Germany. In foot notes are inserted the formulæ which are employed in the different modes of treatment of which the author speaks. This to the young surgeon and even the established practitioner will be found a great convenience and assistance.

The manual of M. Humbert gives an account of the mode of practice adopted by Biett at the hospital of St. Louis in venereal diseases of the skin. The first part of the work notices generally the remedies employed by M. Biett, whilst the second gives a brief description of the several varieties of the "syphilides" as they are termed by Alibert, and the particular indications for the use of each remedy. In these forms of disease the various mercurial preparations are chiefly relied upon by M. Biett in conjunction with vapour-bathing, and attention to the general health. He speaks highly of the muriate of gold and the arseniate of soda in certain cases where mercury had failed. The preparation of mercury on which most reliance is placed is the bichloride. The manual of M. Humbert is a work of great practical value.

---

